# miR-204 is associated with an endocrine phenotype in human pancreatic islets but does not regulate the insulin mRNA through MAFA

**DOI:** 10.1038/s41598-017-13622-7

**Published:** 2017-10-25

**Authors:** Ilaria Marzinotto, Silvia Pellegrini, Cristina Brigatti, Rita Nano, Raffaella Melzi, Alessia Mercalli, Daniela Liberati, Valeria Sordi, Maurizio Ferrari, Massimo Falconi, Claudio Doglioni, Philippe Ravassard, Lorenzo Piemonti, Vito Lampasona

**Affiliations:** 1 Diabetes Research Institute, Division of Immunology, Transplantation and Infectious Disease, IRCCS San Raffaele Scientific Institute, Milan, Italy; 2 Human Pathologies Genomic Diagnostics unit, Division of Genetics and Cell Biology, San Raffaele Scientific Institute, Milan, Italy; 3 Vita-Salute San Raffaele University, Milan, Italy; 4 Department of Surgery, Division of Pancreatic Surgery, San Raffaele Scientific Institute, Milan, Italy; 5 Unit of Pathology, San Raffaele Scientific Institute, Milan, Italy; 6 Institut du Cerveau et de la Moelle épinière (ICM), Biotechnology & Biotherapy Team, Université Pierre et Marie Curie, Paris, France

## Abstract

*miR-204* has been proposed to modulate insulin expression in human pancreatic islets by regulating the expression of the *MAFA* transcript, and in turn insulin transcription. We investigated *miR-204* expression in pancreatic endocrine tumors (PET), a panel of human tissues, tissues derived from pancreatic islet purification, and in induced pluripotent stem cells (iPSCs) differentiated towards a pancreatic endocrine phenotype by quantitative real time RT-PCR or droplet digital PCR (ddPCR). In addition, we evaluated the effect of *miR-204* up- or down-regulation in purified human islets and in the EndoC-βH1 cell line, as an experimental model of human pancreatic β cells. Our results confirm that *miR-204* was enriched in insulin producing PET, in β cells within healthy pancreatic islets, and highly expressed in EndoC-βH1 cells. Moreover, in iPSCs *miR-204* increased stepwise upon stimulated differentiation to insulin producing cells. However, up- or down-regulation of *miR-204* in human islets and in EndoC-βH1 cells resulted in modest and not significant changes of the *MAFA* and *INS* mRNAs measured by ddPCR or c-peptide release. Our data confirm the association of *miR-204* with a β cell endocrine phenotype in human pancreatic islets, but do not support its direct role in regulating the levels of insulin mRNA through MAFA.

## Introduction

The genetic program controlling insulin expression in pancreatic β cells is not fully clarified and a deeper understanding of its functioning holds the potential to find practical applications in the treatment of diabetes.

The discovery of microRNAs (miRNAs), small (20 to 24 nucleotides) noncoding RNA molecules involved in the post-transcriptional control of mRNA translation, constituted a major advance in our understanding of how gene expression is regulated^1^; miRNAs play critical roles in cell proliferation, apoptosis, and development by negatively regulating the stability or translational efficiency of their target mRNAs.

In pancreatic β cells, miRNAs potentially contribute to the pathogenesis of diabetes by acting on cell function and differentiation^2–9^. In a proposed regulatory pathway, *miR-204* has been implicated in the regulation of insulin expression in both human islets and rodent β cell lines^10^, mediated by a direct targeting and downregulation of MAFA, a known insulin transcription factor. In this model, it was reported that TXNIP, a cellular redox regulator implicated in diabetes and pancreatic β cells susceptibility to apoptosis, could indirectly modulate the transcription of *miR-204*. In turn, the levels of *miR-204* were shown to down-regulate the MAFA transcription factor, leading eventually to a reduction in the expression of the insulin gene. The mechanism through which TXNIP exerted this effect was only partially clarified although TXNIP was shown to induce a reduced activity of the transcription factor STAT3, that interacts with the promoter of the TRPM3 gene, within whose intron 6 *miR-204* is encoded^10^.

The aim of this study was to investigate the contribution of *miR-204* to the regulation of the insulin gene transcript; we analyzed *miR-204* and insulin expression in pancreatic endocrine tumors (PET), induced pluripotent stem cells (iPSCs) in the course of their differentiation towards an insulin producing endocrine phenotype, human islets, and in the human EndoC-βH1^11^ cell line, as a model of pancreatic β cells.

## Results

### Expression of *miR-204* is associated with an insulin functional phenotype in PETs

**Figure 1:**
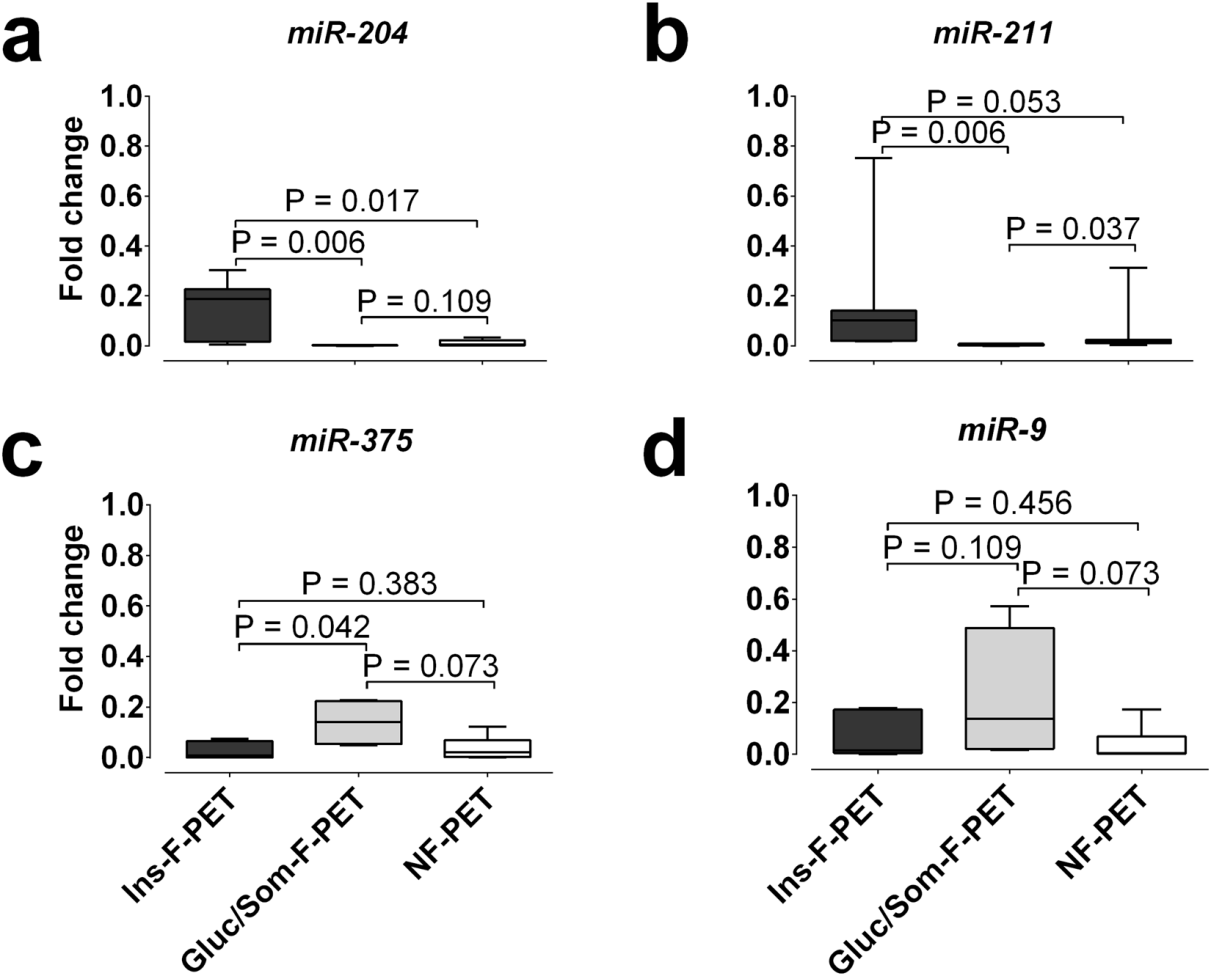
*miR-204* and selected miRNA expression in PET. Box and whisker plot (min to max) of *miR-204*, *miR-211*, *miR-375*, and *miR-9* levels expressed as fold change relative to median levels in human islets (HI) in Ins-F-PET (dark grey boxes), Gluc/Som-F-PET (light grey boxes), and NF-PET (clear boxes); the significance of differences was analyzed using the Mann Whitney test.

Expression of *miR-204* and of the closely related *miR-211*, *miR-375*, and *miR-9* was analyzed by real-time qRT-PCR in functional pancreatic neuroendocrine tumors (PET) of which 7 expressed insulin (Ins-F-PET), 4 glucagon or somatostatin (Gluc/Som-F-PET, 3 glucagonomas, 1 somatostatinoma), and in 7 non-functional tumors (NF-PET) (Supplementary Table S1). The comparison of Ins-F-PET with NF-PET and with Gluc/Som-F-PET identified *miR-204* and *miR-211* as significantly over-expressed in insulinomas (Fig. 1). Logistic regression analysis, based on negative or positive immunohistochemical staining, showed that in PETs the expression of insulin at the protein level was predicted by both *miR-204* (OR: 16.8, 1.49–189 p = 0.022) and *miR-211* (OR: 9.65, 1.09–85 p = 0.041) but not by *miR-375* (OR: 0.35, 0.09–1.34) or *miR-9* (OR: 0.73; 0.23–2.26). A consistent trend for higher expression of *miR-375* and *miR-9* in Gluc/Som-F-PET was also evident. *miR-204* and *miR-211* expression levels were not related to patient age, sex, tumor size, tumor location (head vs body-tail), tumor proliferation index, tumor histological classification (well-differentiated endocrine neoplasms vs carcinomas). Linear regression analysis showed that *miR-204* expression in all PET sub-groups correlated with expression of its homologous *miR-211* (Supplementary Fig. S1), likely as a consequence of partial cross-reactivity between the *miR-204* and the *miR-211* assays. The specificity of *miR-204/211* for Ins-F-PET was confirmed by the presence of a statistically significant positive correlation of *miR-204/211* and insulin but not glucagon mRNA levels (Supplementary Fig. S2). The mRNA expression of insulin, glucagon and transcription factors involved in pancreatic development (*PDX1, NKX6.1, NKX2.2, PAX4, PAX6, ONECUT1, NGN3, ISL1, PTF1A, NEUROD1*) was determined in PETs where sufficient material was available using qRT-PCR (Supplementary Fig. S3). Neither the expression of *miR-204* or *miR-211* nor that of *miR-375* and *miR-9* correlated with any of the other evaluated genes (Supplementary Table S2). All together these data demonstrate a correlation between *miR-204/211* and insulin expression at both the mRNA and protein level in PET.

### *miR-204/211* are expressed in different human tissues and, in the pancreas, are specifically enriched in islets

**Figure 2:**
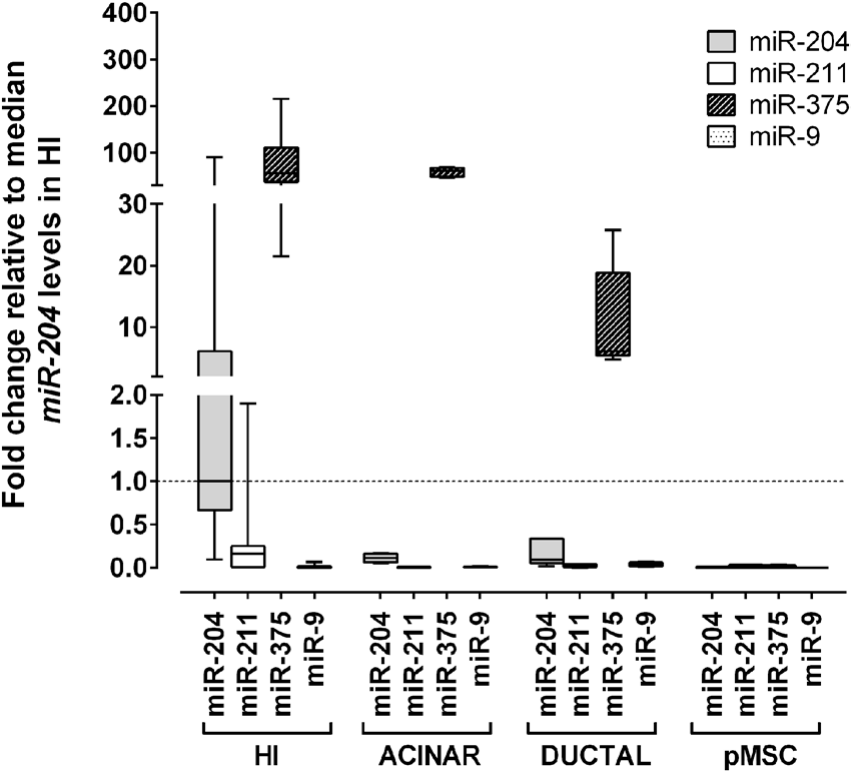
miRNA expression in cells derived from purified human pancreatic islets (HI). Box and whisker plot (min to max) of *miR-204* (grey boxes), *miR-211* (clear boxes), *miR-375* (dark striped boxes), and *miR-9* (dotted boxes) levels in human islets (HI), acinar, ductal, and pMSC cells. Expression was measured by qRT-PCR and is reported as fold change relative to median miR-204 levels in HI (dashed line).

Among normal tissues, the expression of *miR-204* was highest in the kidney, followed by brain, testes, ovary, and then pancreatic islets (Supplementary Fig. S4) with levels just below the 3rd quartile of all tissues tested. Expression of *miR-211*, while overall lower, was highest in the brain and then in islets. Expression of *miR-204/211* was detectable in all islet preparations tested, with a predominance of *miR-204* whose median levels were >10 fold higher compared to *miR-211* (Fig. 2). The levels of both miRNAs also differed widely between islet preparations, in particular *miR-204* observed levels showed a >100-fold change from lowest to highest value. Expression of *miR-204* was clearly higher in purified islets compared to either acinar or ductal enriched cell fraction obtained after purification, with median levels 8.9 and 11.5 fold greater, respectively. Expression of *miR-211* was also strongest in purified islets compared to either acinar or ductal cells, with median levels 38.5 and 11.5 fold greater, respectively. These data suggest that in pancreatic islets both *miR-204* and *miR-211* are predominantly enriched in endocrine cells. Compared to *miR-375*, the most abundant miRNA in pancreatic islets, *miR-204* and *miR-211* median levels were 30 and 277 fold lower, respectively. The variability of expression of *miR-375* in islets was less pronounced than that of *miR-204* with a 10-fold change from the lowest to highest observed value. Levels of both *miR-204* and *miR-375* showed a trend towards a positive correlation with the degree of islet purity although statistically not significant (*miR-204* Spearman R = 0.62, p = 0.14; *miR-375* Spearman R = 0.68, p = 0.11). In pancreatic islets expression of *miR-9*, another miRNA implicated in the regulation of insulin secretion, was markedly lower compared to both *miR-204* and *miR-375*, with median levels 87 and 3857 fold lower, respectively.

### *miR-204* is upregulated when iPSCs are differentiated towards a β cell-like phenotype

The association of *miR-204* and *miR-211* expression with the acquisition of a β cell-like phenotype was tested in the course of iPSCs differentiation towards pancreatic endocrine cells^12^. Differentiation of 3 different human iPSCs preparations was induced using a protocol previously adopted for hESCs with modifications, resulting in the generation of a mean of 5% of insulin positive cells (data not shown), in line with previously described results^13^. Expression of *miR-204* and *miR-211* together with that of the *INS*, *NGN3* and *PDX1* genes was determined at sequential time-points corresponding to differentiation stages of the endocrine pancreas (Supplementary Fig. S5). With respect to basal levels in undifferentiated iPSCs, expression of *miR-204*, *INS*, and *PDX1* during differentiation from the DE to the EN stages reaching a median increase of 6, 51, and 2 × 10^6^ folds, respectively. The *NGN3* mRNA instead increased upon induction of differentiation but showed a different expression profile with a 20-fold peak at the PE stage. Expression levels of *miR-211* both in basal or differentiated iPSCs were below detection limits.

### *miR-204* quantification by ddPCR

**Figure 3:**
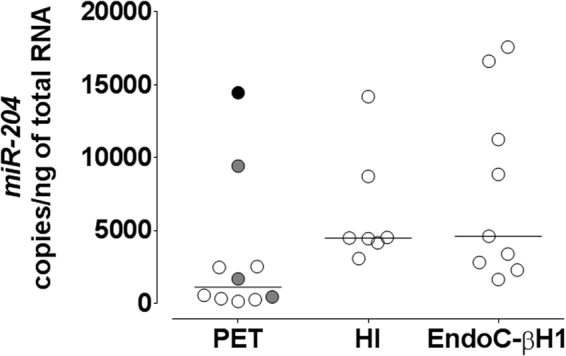
ddPCR quantification of *miR-204* expression in PET, Human Islets and EndoC-βH1. Scatter plot of *miR-204* copies per ng of total RNA measured in ddPCR from 10 pancreatic endocrine tumors (PET: 1 Ins-F-PET black circle, 3 Gluc/Som-F-PET grey circles, 6 NF-PET clear circles), 7 purified Human Islets preparations (HI), and 9 samples from EndoC-βH1 cells at different time points in culture.

Considering the clear predominance in normal purified human islets of *miR-204* relative to *miR-211*, we decided to further characterize exclusively *miR-204*. We assessed the feasibility to study the function of *miR-204* in pancreatic β cells using droplet digital PCR (ddPCR) for the precise and absolute quantification of selected miRNA and gene transcripts in a selection of PET (n = 10), purified human islets preparations (n = 7), and in the recently established β cell line of human origin EndoC-βH1. Total RNA was extracted using a procedure that preserves the content of small RNAs and subjected to reverse transcription followed by ddPCR. *miR-204* was detected in all the analyzed PET, with a median number of copies per ng of total RNA of 1123 (range 142–14447) (Fig. 3). The largest amounts of *miR-204* were found in the single insulinoma tested and in human islet preparations, with a median of 4488 copies/ng of total RNA (range 3079–14182). *miR-204* was also clearly expressed in the human β cell line EndoC-βH1, with a median of 4612 copies/ng of total RNA (range 1642–17576) measured in 9 RNA preparations collected at different time points in culture.

### Correlation in human islets of *miR-204*, *MAFA*, *INS*, *TXNIP*, and *TRPM3* mRNA levels measured by ddPCR

**Figure 4:**
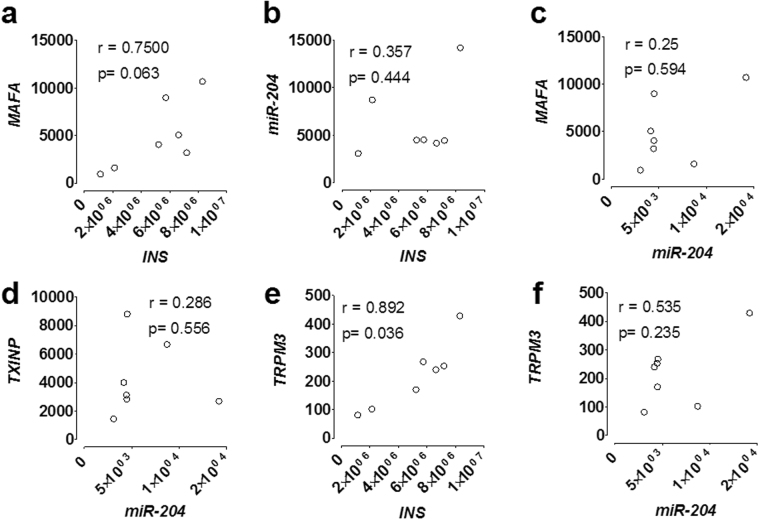
Correlation in Human Islets of *miR-204*, *MAFA*, *INS*, *TXNIP*, and *TRPM3* mRNA levels measured by ddPCR. XY plots of the corresponding transcript levels in 7 Human Islets preparations measured by ddPCR and expressed as copies per ng of total RNA. Shown are the corresponding Spearman r and p values. The only significant correlation observed (*TRPM3* vs *INS*, panel 4e) was corrected for multiple comparisons.

In purified human islets *INS* mRNA levels showed a trend towards a partial correlation with those of the *MAFA* transcripts, although it did not reach statistical significance, (Spearman r = 0.75, p = 0.063) (Fig. 4a), and no correlation with those of *miR-204* (Spearman r = 0.357, p = 0.444) (Fig. 4b). No correlation was observed also between *MAFA* (Spearman r = 0.25, p = 0.594) (Fig. 4c) or *TXNIP* (Spearman r = 0.286 p = 0.556) (Fig. 4d) and *miR-204* transcripts levels. Instead, the *INS* mRNA showed a positive correlation with that of *TRPM3* (Spearman r = 0.892, p = 0.036 corrected for multiple comparison) (Fig. 4e). Correlation of *miR-204* with *TRPM3* was instead only partial and did not reach statistical significance (Spearman r = 0.535, p = 0.235) (Fig. 4f).

### Effect of up- or down-regulation of *miR-204* in Human Islets on *INS*, *MAFA*, *TXNIP*, and *TRPM3* mRNA levels

**Figure 5:**
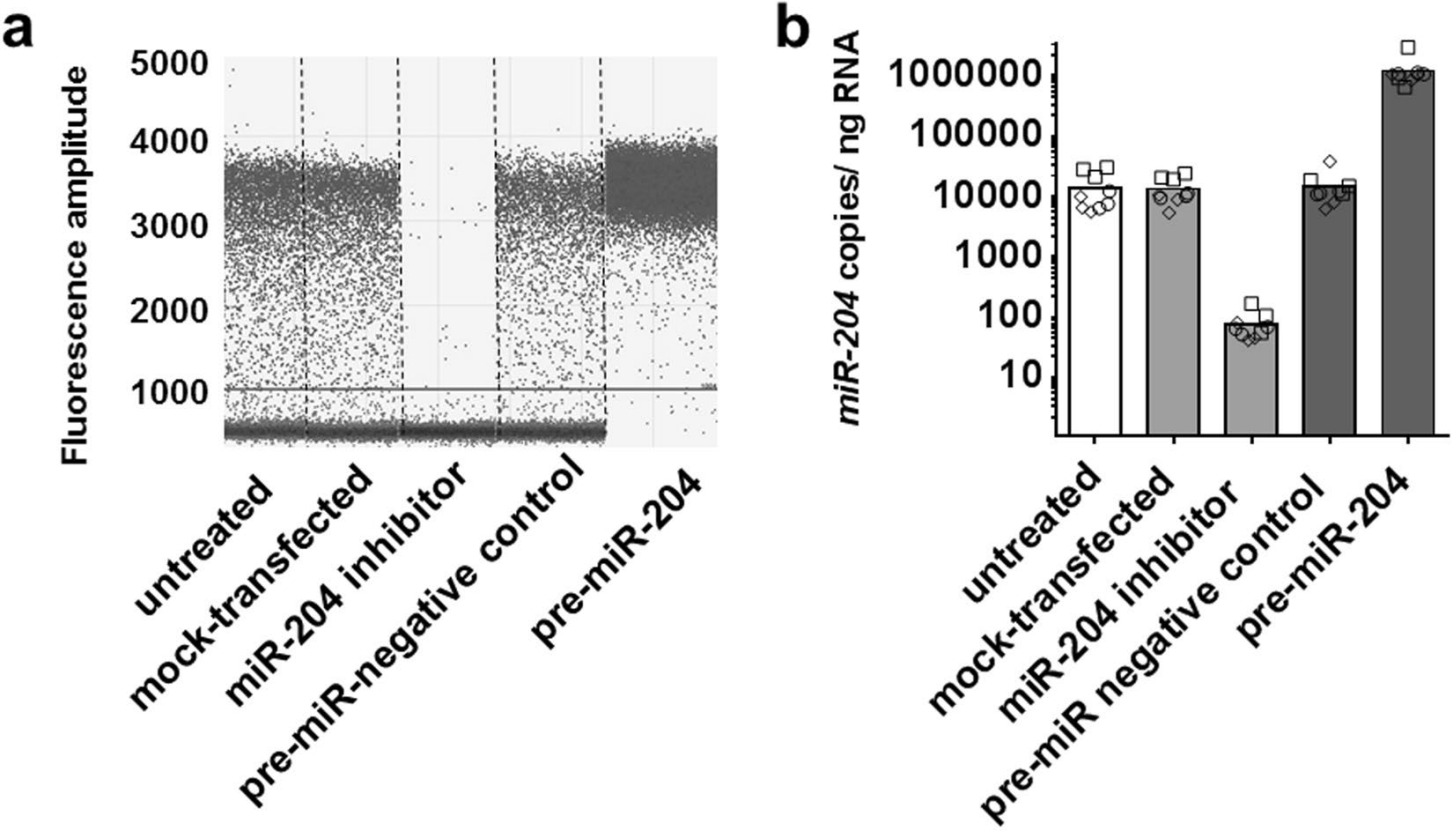
*miR-204* up- and down-regulation in Human Islets. Scatter plot of ddPCR droplets’ fluorescence results from a representative experiment of *miR-204* up- or down-regulation in transfected purified human islets. Vertical dotted lines separate treatment groups (panel a). Bar plot of mean *miR-204* copies per ng of total RNA measured by ddPCR, according to treatment group. Individual results of 3 transfection replicates are shown for each islet preparation (squares = HP1260, circles = HP1266; diamonds = HP1269). Treatment groups are identified by bar color: untreated cells white bars, *miR-204* down-regulation light grey bars, *miR-204* up-regulation dark grey bars (panel b).

**Figure 6:**
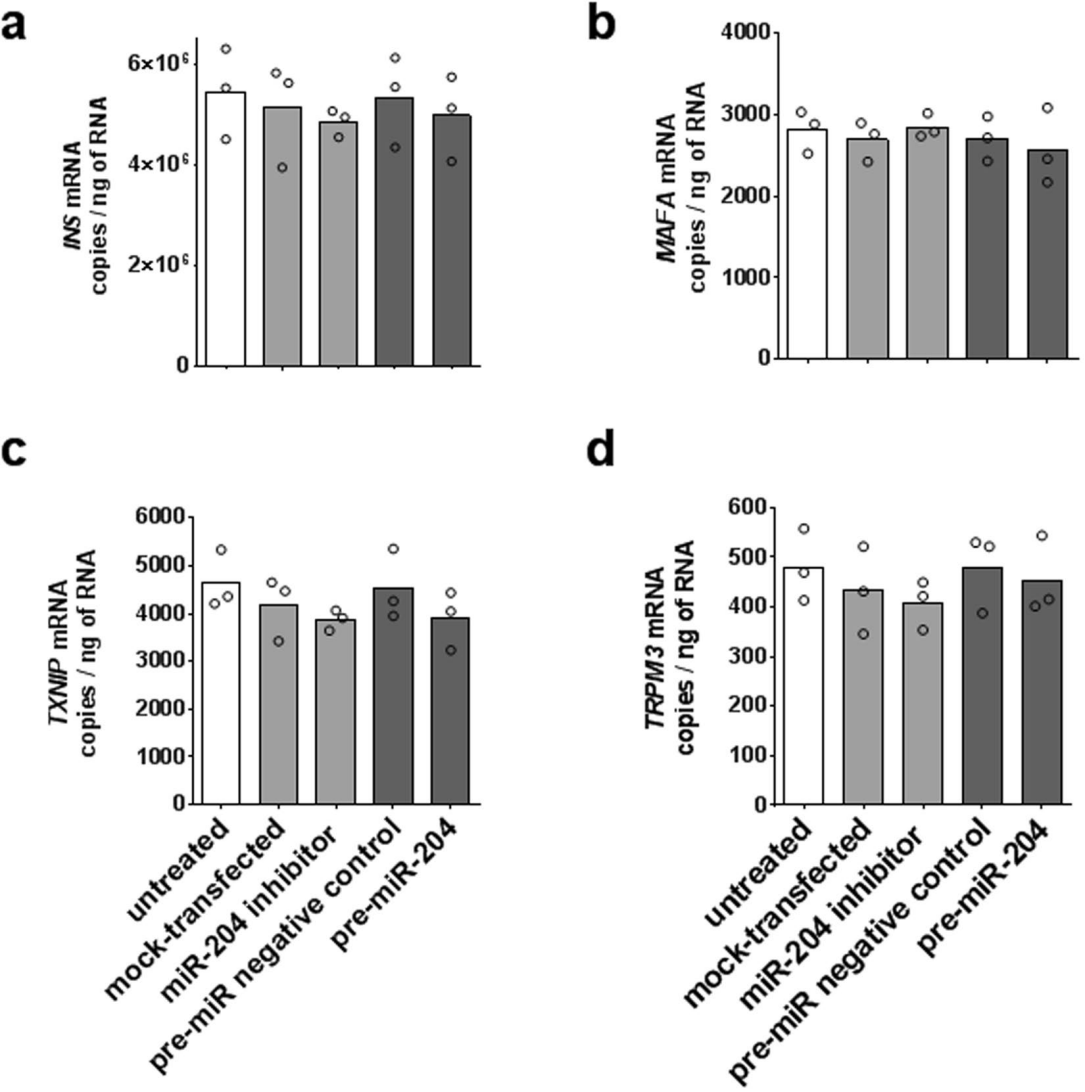
Effect of *miR-204* up- or down-regulation on *INS*, *MAFA*, *TXNIP*, and *TRPM3* transcripts in isolated human islets. Bar plots of average copies per ng of total RNA measured by ddPCR for the *INS*, *MAFA*, *TXNIP*, and *TRPM3* (panel a,b,c,d, respectively) in *miR-204* up- and down-regulation experiments in the HP1260 human islets preparation. Individual results of 3 transfection replicates are shown. Treatment groups are identified by bar color: untreated cells white bars, *miR-204* down-regulation light grey bars, miR-204 up-regulation dark grey bars.

Three independent experiments were performed in which *miR-204* was up- or down-regulated in pancreatic human islets from different cadaveric donors (HP1260, HP1266, HP1269) and *INS*, *MAFA*, *TXNIP*, and *TRPM3* transcripts subsequently measured by ddPCR. For *miR-204* up-regulation, purified human islets were dissociated and transfected with a *pre-miR-204* precursor or, for comparison, with an irrelevant scrambled miRNA precursor. For *miR-204* down-regulation, the same islets were transfected with a *miR-204* hairpin inhibitor or with transfection reagent alone as a mock treatment. In each experiment, each treatment was performed in in three parallel transfection replicates and the viability of islets at the end of treatment was assessed by flow cytometry. In all experiments, treatments induced a substantial degree of cell death at the end of the 72 hours incubation post-transfection, with a viability of 87% (HP1260, range 83–90%), 54% (HP1266, range 46–58%), and 25% (HP1269, range 16–34%), respectively. Cell death was not significantly associated with a specific treatment in any of the islets preparations. Overall, a 72-hour incubation with the *miR-204* precursor induced an 85 fold mean increase of *miR-204* compared to islets transfected with a scrambled miRNA, while transfection with a *miR-204* hairpin inhibitor resulted in a 167 fold mean decrease of *miR-204* compared to mock transfected islets (Fig. 5). In the HP1260 islet preparation, that showed the highest viability post transfection, the INS transcript displayed a 5% mean decrease upon *miR-204* up-regulation and a 6% mean decrease upon *miR-204* down-regulation compared to the appropriate control treatment (Mann Whitney test p = 0.7 for both) (Fig. 6a). For the *MAFA* transcript, we observed a modest and statistically not significant 5% decrease of copy number upon *miR-204* upregulation (Mann Whitney test p = 1.0) and a 6% mean increase when *miR-204* was down-regulated (Mann Whitney test p = 0.7) (Fig. 6b); the *TXNIP* transcript showed a 14% mean decrease when *miR-204* was up-regulated (Mann Whitney test p = 0.222) and a 7% mean decrease when *miR-204* was down-regulated (Mann Whitney test p = 0.7 for both) (Fig. 6c); the *TRPM3* transcript showed a 5% mean decrease when *miR-204* was up-regulated (Mann Whitney test p = 0.895) and a 6% mean decrease when *miR-204* was down-regulated (Mann Whitney test p = 1.0 for both) (Fig. 6d). Similar results were obtained in islet preparations with reduced viability (Supplementary Fig. S6). Overall, while the results showed wide variations in the copy number of the studied mRNAs across islets preparations, we did not observe significant changes in the levels of any of the analyzed transcripts when *miR-204* was either up- or down-regulated compared to control treatments.

### Effective up- and down-regulation of *miR-204* by transfection of *miR-204* precursor or hairpin inhibitor in the human β cell line EndoC-βH1

We investigated the effect of *miR-204* up- or down-regulation in the EndoC-βH1 cell line, as a model of pancreatic β cells. We performed 4 experiments, each comprising 3 parallel transfections for all treatments, in which *miR-204* and *INS*, *MAFA*, *TXINP*, *TRPM3*, and *GAPDH* transcripts were measured by ddPCR at 72 (1 experiment) and 48 (3 experiments) hours post transfection. At 72 hours, we observed a 135 fold increase of *miR-204* upon transfection with the *pre-miR-204* precursor compared to an irrelevant scrambled miRNA precursor, while transfection with a *miR-204* specific hairpin inhibitor instead reduced *miR-204* by 49 folds relative to mock-transfected cells (Supplementary Fig. S7). Experiments in which EndoC-βH1 cells were incubated for 48 hours post transfection showed similar results (Supplementary Fig. S8), with a mean 144 fold increase (Mann Whitney test p < 0.0001) and a mean 171 fold decrease (Mann Whitney test p < 0.0001) of *mir-204* upon up- or down-regulation, respectively.

**Figure 7:**
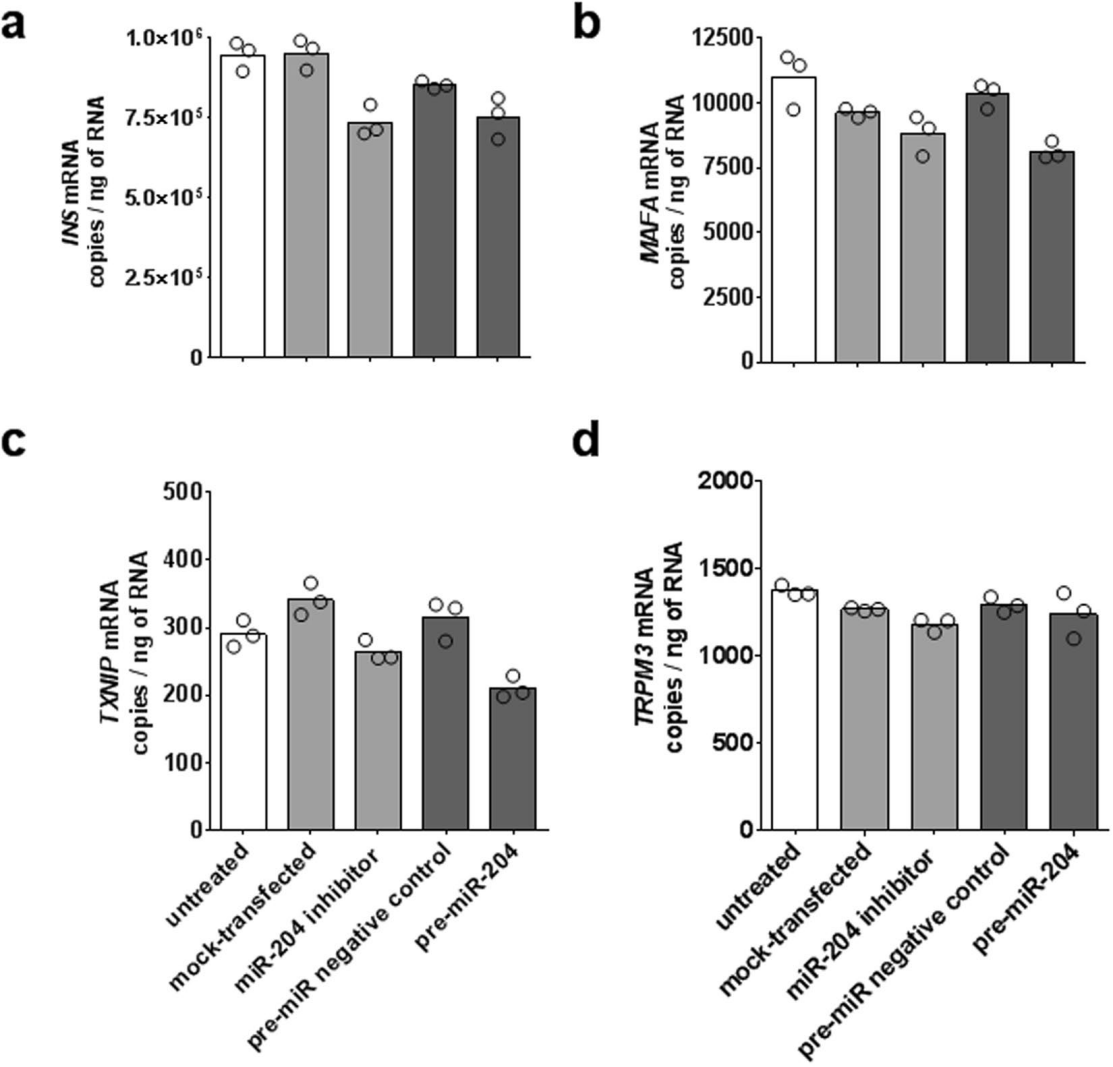
Effect of *miR-204* up- or down-regulation on *INS*, *MAFA*, *TXNIP*, and *TRPM3* transcripts in EndoC-βH1. Bar plots of average copies per ng of total RNA measured by ddPCR for the *INS*, *MAFA*, *TXNIP*, and *TRPM3* (panels a,b,c,d, respectively) in *miR-204* up- and down-regulation experiments. Individual results of each transfection replicate performed in parallel (n = 3) are shown as circles. Treatment groups are identified by bar color: untreated cells white bars, *miR-204* down-regulation light grey bars, *miR-204* up-regulation dark grey bars.

At 72 hours post transfection, the copies per ng of total RNA of the *INS* transcript showed a 12% decrease in cells transfected with the *pre-miR-204* precursor and a 13% decrease in cells transfected with a *miR-204* specific inhibitor compared to the appropriate control treatment (Fig. 7a). Neither effect reached statistical significance (Mann Whitney test p values equal to 0.1 for both). Copies of the *MAFA* transcript showed a 21% decrease when *miR-204* was up-regulated and a 9% decrease when *miR-204* was down-regulated; both changes did not reach statistical significance (Mann Whitney test p values equal to 0.1 and 0.2, respectively) (Fig. 7b). Copies of the *TXNIP* transcript showed a 33% decrease when *miR-204* was up-regulated and a 23% decrease when *miR-204* was down-regulated (Mann Whitney test p values equal to 0.1 for both) (Fig. 7c). The transcript of the *TRPM3* gene, within whose intron 6 the *miR-204* gene is encoded, showed a 4% decrease when *miR-204* was up-regulated and a 7% decrease when *miR-204* was down-regulated (Mann Whitney test p values equal to 0.9 and 0.1, respectively) (Fig. 7d). In the three experiments in which mRNA levels were measured 48 hours post transfection, we observed similar not significant results for the *INS* and *TRPM3* transcripts while, when *miR-204* was up-regulated, *MAFA* and *TXNIP* transcripts showed a mean decrease of 32% and 40% respectively (Mann Whitney test p values equal to 0.0005 and 0.0002) (Supplementary Fig. S9).

As an additional analysis, to evaluate discrepancies between absolute mRNA ddPCR measurement and quantification relative to a housekeeping gene, we compared mRNAs’ fold changes with or without normalization of copy number relative to the levels of the *GAPDH* transcript. In both the 72 and 48 hours incubation experiments, this analysis showed a high degree of correlation of the fold change normalized by *GAPDH* results with those directly based on copy number per ng of total RNA (Spearman r equal to 0.7349, p = 0.0458, and to 0.8910, p < 0.0001, respectively) (Supplementary Fig. S10).

### Effect of up- or down-regulation of *miR-204* in EndoC-βH1 on C-peptide secretion

**Figure 8:**
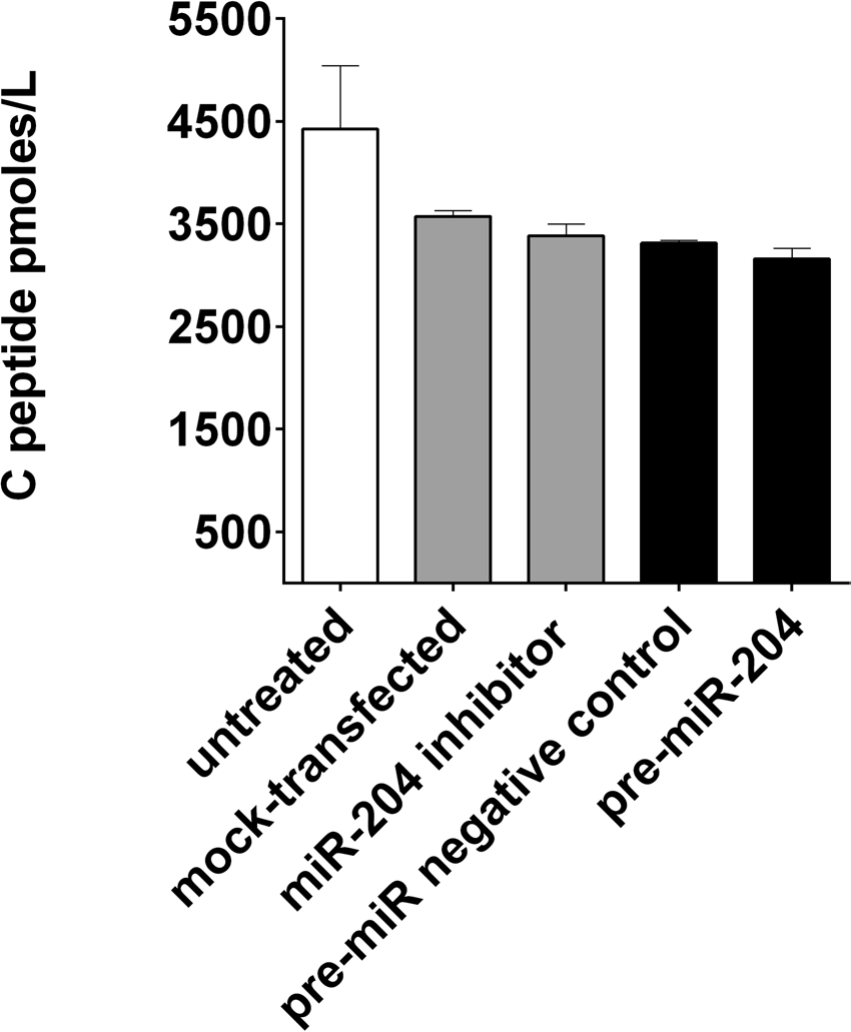
Effect of *miR-204* up- or down-regulation on c-peptide release in EndoC-βH1 supernatants. Bar plot of picomoles/liter of c-peptide in culture supernatant measured by ELISA at day 1 after *miR-204* up- and down-regulation. Results are shown as mean + SD of two technical replicates. Treatment groups are identified by bar color: untreated cells white bars, *miR-204* down-regulation light grey bars, *miR-204* up-regulation dark grey bars.

C-peptide content in culture supernatants of EndoC-βH1 was measured by ELISA at day 1 and 2 after up- or down-regulation of *miR-204*. Compared to untreated cells a not significant reduction in c-peptide content was observed in all treatment groups. No significant changes were observed when *miR-204* was either up- or down-regulated compared to mock transfected cells or cells transfected with an irrelevant negative control miRNA (Fig. 8).

### The *miR-204* target sequence in the 3′ UTR of the human *MAFA* transcript is not canonical

A bioinformatics analysis of potential mRNA targets of *miR-204* was performed with a panel of online tools based on alternative algorithms: miRanda (complementary method), TargetScan (seed-complementary method), DIANAmicroT (thermodynamics method), RNAhybrid (Thermodynamics & Statistical model method), and MiRtaget2 (Support Vector Machine method)^14–18^. None of these alternative algorithms identified either *TXNIP* or *MAFA* as targets of human *miR-204*. In addition, compared to mouse sequences, the human *miR-204* (NCBI Refseq NR_031919.1) and *MAFA* 3′ UTR (NCBI Refseq NM_201589.3) present an additional single nucleotide mismatch within the miRNA seed sequence, suggesting that the interaction of *miR-204* and *MAFA* is non canonical (Supplementary Fig. S11).

## Discussion

The acquisition and maintenance of an endocrine phenotype by normal pancreatic islets has been associated with the action of miRNAs, a powerful class of post-transcriptional regulators of gene expression^2–4,19^. Insulin expression itself is critically dependent on miRNAs’ activity, as shown by its dramatic down-regulation upon conditional deletion of Dicer in β cells^20^. Moreover, several miRNAs, like *miR-375*, *miR-124a*, *miR-96*, and *miR-9*, are implicated in the regulation of insulin secretion^5,6,21,22^.

In this light, we found of particular interest the novel pathway recently proposed by Xu *et al*. in which an additional miRNA, *miR-204*, influenced insulin mRNA levels by exerting a direct action on the mRNA of *MAFA*, a known insulin gene transcription factor^10^.

Older studies that analyzed the miRNA transcriptome in the pancreas or in islets failed to identify *miR-204*, or the closely related *miR-211*, as enriched in pancreatic tissue. Several factors might have contributed to this lack of detection of *miR-204/211*, including known technical differences in RNA extraction, detection systems, and normalization strategies, that can profoundly affect miRNA discovery and quantification^23–26^. In addition, it is worth remembering that islets constitute less than 5% of the pancreas mass, making potentially difficult the detection of endocrine specific miRNAs expressed at intermediate to low levels or showing a relatively large variability between islets preparations. However, the expression of *miR-204* was previously reported as restricted to insulinomas in pancreatic endocrine tumors (PET)^27^ and enriched in β cells compared to α cells in purified pancreatic islets^28^, suggesting a potential role in either β cell differentiation or insulin expression regulation or both.

In this report, we studied the relationship between insulin mRNA and *miR-204* levels, both in PET, normal purified human islets, and in a human β cell line model. Consistent with previous reports, *miR-204* expression was enriched in insulinomas and drastically lower in both non-functional PETs and functional PETs expressing glucagon or somatostatin. Moreover, *miR-204* was also clearly detectable in healthy purified islets, with levels higher than in PETs or co-purified non-endocrine components like acinar, ductal cells, and mesenchymal stem cells resident within islets, despite the known ability of the latter to up-regulate *miR-204* under suitable conditions^29^. However, we also observed that *miR-204* levels, while clearly increasing in the course of the induced differentiation of iPSCs towards endocrine cells, did not strictly correlate with the acquisition of a terminal differentiated β cell phenotype. Overall, these observations support the concept that, within normal pancreatic islets, *miR-204* is likely to be enriched in the β cells of mature islets and related to some unspecified β cell functions.

Surprisingly, our study failed instead to replicate the main observations linking *miR-204* to the regulatory pathway of the *INS* gene expression mediated by MAFA. Unlike in the original report, in our hands a major up- or down-regulation of *miR-204* reduced *MAFA* levels only under some experimental conditions and did not result in significant changes of the *INS* mRNA. This discrepancy might be explained by several factors, that include the wide variability of the insulin mRNA levels in human islets preparations under stress conditions, the possible aspecific interactions of miRNA under non-physiological conditions, the choice of a human rather than a murine cell line as model, and finally the adoption in previous studies of real-time PCR techniques ill-suited to precisely and reliably measure small relative differences.

In our study, we have repeatedly observed a large variability in the amount of miRNA and insulin transcripts found in different batches of purified human islets or even in the same human β cell line at different time points in culture, using both conventional real-time PCR relative quantification and droplet digital PCR absolute quantification. Irrespective of whether this variability in insulin mRNA content reflects a true biological difference, originally present between islets preparations, or a response to physiological stimuli in culture, or more simply an effect of cellular stress, like that of the isolation and dissociation procedure in the case of purified islets, these differences clearly exceeded those previously reported as the product of the artificial modulation of *miR-204* ^10^. This suggests that the *INS* mRNA, while always very abundant in absolute terms, is subjected to ample fluctuations in islets and β cell lines, and implies that this natural variability can exert a potentially important confounding influence. Moreover, the modulation of *miR-204*, performed according to the originally published protocol, resulted in massive changes of *miR-204* levels that were far larger than any of those measured across untreated islets batches or cell lines. It is therefore questionable whether the changes in insulin mRNA levels upon *miR-204* modulation previously reported could be representative of what occurs in untreated β cells where the physiological levels of *miR-204* are consistently orders of magnitude lower.

In addition, the use of a rodent cell model for part of the experiments conducted in the original report, introduces another potential confounding factor due to the presence of differences between human and rodent genomic sequences. In fact, the proposed seed sequence of *miR-204* in the 3′ UTR region of the rat *MAFA* transcript contains a nucleotide mismatch in humans, therefore classifying the potential interaction of *miR-204* and *MAFA* in humans as non-canonical. As typical for most miRNAs, also for *miR204* the prediction *in silico* of the pathways in which it could be potentially involved leads to multiple alternatives of which only a fraction has been experimentally validated and only in tissues other than islets^27,29–31^. However, while it is unclear how any of these predicted or described pathways could be linked to the maintenance and acquisition of an endocrine phenotype, it is of notice that none of the proposed interactions between *miR-204* and *MAFA*, could be predicted by any of the algorithms that we applied to identification of *miR-204* targets.

While the mismatch present in the candidate human seed sequence could explain the negligible or minor effect of *miR-204* up- or down-regulation on the transcript levels of MAFA that we observed in our study, the current understanding of miRNAs’ function allows for translational repression, also following non-canonical interactions^32^, that are independent of the downregulation at the transcript level of the miRNAs’ targets.

However, while *miR-204* might therefore theoretically lead to reduced levels of the MAFA protein in the absence of a significant effect on its transcript, we could not replicate in both purified human islets and the EndoC-βH1cell line the originally suggested downstream effect of *mir-204* up-regulation and subsequent MAFA protein decrease i.e. the reduction of the insulin transcript levels.

Of notice, in the original report the assessment of *INS* and other genes mRNA levels was based on classical real-time PCR relative quantification. Conventional qPCR has known limitations when aimed at determining relative differences in RNA content as small as those previously described as the consequence of *miR-204* up our down-modulation^10^. To produce robust data this would have required an understandably unwieldy number of technical replicates^33,34^ which, unfortunately, is rarely if ever found in most publications based on conventional qPCR. In our study, to reduce the issues of reliability, reproducibility, and precision when measuring changes of mRNA levels upon *miR-204* modulation, we opted instead for the use of droplet digital PCR, a state of the art technique for precise absolute quantification of nucleic acids^35^.

Overall, the evidences we have collected indicate that the proposed *miR-204* > MAFA > INS pathway might not be relevant in human pancreatic β cells. Although the mechanisms through which *miR-204* might be linked to the endocrine phenotype in the pancreas and β cell identity in islets await clarification, we are reporting the observation of a potential correlation between the levels of the insulin mRNA and those of the transient receptor cation channel TRPM3 in the EndoC-βH1 model. TRPM channels in islets are thought to be key players in the regulated release of insulin^36,37^ and TRPM3 in particular is known to mediate the regulated uptake of zinc from the extracellular space mouse β cells^38^, when engaged by the neurosteroid hormone pregnenolone sulphate, to potentiate insulin secretion^39^, and activate an Erg-1 dependent transcriptional pathway leading to PDX1, and in turn insulin, up-regulation^40^. Furthermore, in a clear cell renal cell carcinoma model^41^, *mir-204* post-translationally regulates TRPM3, eliciting a coordinated function in pathways in which TRPM3 is involved.

Further studies will be required in order to unravel the question if in islets the expression of *miR-204* is merely reflective of that of *TRPM3* or has an additional role independent from that of its host gene.

## Methods

### Primary human tissues

Pancreatic endocrine tumors (PET) samples were collected at the San Raffaele hospital (Milan, Italy) after approval of the study by the San Raffaele Scientific Institute ethics committee in accordance with local guidelines and national regulations of the Italian Ministry of Health. In accordance with the principles of the Declaration of Helsinki as revised in 2000, written informed consent was obtained from all adult participants before biopsy collection; 18 tumors were used in qRT-PCR experiments, of these 11 were functional hormone expressing tumors (F-PET) including 7 insulinomas (Ins-F-PET), 3 glucagonomas, 1 somatostatinoma (Gluc/Som-F-PET), and 7 non-functional tumors negative for all hormones (NF-PET) (Supplementary Table S1); an additional 10 PET samples (1 Ins-F-PET, 3 Gluc/Som-F-PET, and 6 NF-PET) were used in ddPCR experiments. Human pancreatic islets preparations were isolated from 10 heart-beating cadaveric organ donors as previously described^42^, their use for scientific research was approved by the San Raffaele Scientific Institute ethics committee^43^ in accordance with local guidelines and national regulations of the Italian Ministry of Health. Islet purity was assessed as the percentages of endocrine clusters positive to dithizone staining (median purity achieved = 66%, range 30% to 90%). Acinar enriched tissue or ductal cells were obtained from the islet-depleted fraction after pancreatic islet purification according to standard protocols^44^. Briefly, 2 ml of pellet was seeded in suspension tissue flasks and cultured for 12 days in Ham’s F12 (Lonza, Basel, Switzerland) supplemented with 1% BSA (Sigma-Aldrich, St. Louis, MO, USA), 1% Pen/Strep and 1% L-glutamine (Lonza). Ductal phenotype was defined as double positivity in FACS analysis for the Ca19.9 (Acris Antibodies, San Diego, CA) and EpCAM (R&D system, Minneapolis, MN, USA) markers. Pancreatic mesenchymal stem cells (pMSC) were obtained and cultured as previously described^45^; pMSC were recovered from the dense fraction remaining after the isolation of islet cells and grown in α-MEM (Lonza) supplemented with 10% FBS (Lonza) until confluence, when their mesenchymal phenotype was confirmed by checking for co-expression of the CD73, CD90, and CD105 markers (R&D systems, Minneapolis, MN, USA).

### Cell lines and iPSCs

The genetically engineered human pancreatic β cell line EndoC-βH1^11^ was grown in DMEM low glucose (1 g/L) (Gibco, Thermo Fisher Scientific, Waltham, MA, USA), 2% BSA (Sigma), 50 μM 2-mercaptoethanol (Sigma), 10 mM nicotinamide (Sigma), 5.5 μg/mL transferrin (Gibco), 6.7 ng/mL sodium selenite (Sigma), penicillin (100 U/mL) (Sigma), and streptomycin (100 μg/mL) (Sigma). iPSCs obtained from human fetal fibroblasts were kindly provided by V. Broccoli (Division of Neuroscience, IRCCS San Raffaele Scientific Institute, Milan, Italy) and differentiated using a five-step protocol previously validated in human embryonic stem cells (hESC)^13^, with slight modifications^12^, to hormone-expressing endocrine cells through a series of endodermal intermediates resembling those that occur during pancreatic development *in vivo*: definitive endoderm; posterior foregut; pancreatic endoderm; hormone expressing endocrine cells.

### *miR-204* up- and down-regulation in purified human islets

For *mir-204* up or down regulation experiments human pancreatic islets from 3 different donors were used HP1260, HP1266, and HP1269 (median islets purity = 80%, range 70% to 80%). Purified islets were dissociated using TrypLE™ Express (Gibco, Thermo Fisher Scientific) following the manufacturer’s instructions. Single cells were resuspended in Final Wash/Cultured Medium (Mediatech Inc., Herndon, VA) supplemented with 1% L-glutamine (Lonza) and seeded at 1 × 10^6^ cells/well in the wells of a not coated 6 wells plate, to avoid cell adhesion. Dissociated islets were then transfected using the DharmaFECT1 transfection reagent (Dharmacon, Lafayette, CO, USA) according to the manufacturer’s instructions. In *miR-204* up-regulation experiments, cells were transfected with either the *hsa-miR-204* specific Pre-miR™ precursor or the Pre-miR™ negative control 2 (Life Technologies), a random sequence Pre-miR™ molecule validated to produce no identifiable effects on known miRNA function, at a final concentration of 25 nM^10^. In *miR-204* down-regulation experiments, cells were transfected with the miRIDIAN hairpin inhibitor *rno-miR-204* (Dharmacon) at a final concentration of 25 nM^10^. Cells were then cultured for 72 hours before harvest and RNA extraction. In each experiment triplicates transfections were performed in parallel for each precursor, inhibitor, and control treatment. RNA from each transfection replicate was then independently extracted, subjected to reverse transcription in duplicate, and then quantified by ddPCR again in duplicate technical replicates for each RT reaction. Results were then expressed as mean copies per ng of total RNA for each transcript after pooling ddPCR technical duplicates.

### *miR-204* up- and down-regulation in EndoC-βH1 cells

EndoC-βH1 cells were seeded at 2 × 10^5^ cells/well in a 6 wells plate, cultured for 1 week, and then subjected to *miR-204* up- and down-regulation as described above for purified islets, allowing the transfection to proceed for either 48 (three independent experiments) or for 72 hours (one experiment).

### RNA isolation and gene expression analysis by real-time qRT-PCR

Total RNA was isolated using the mirVana miRNA isolation kit (Life Technologies, Milano, Italy) or the NucleoSpin miRNA kit (Macherey-Nagel, Düren, Germany) following the manufacturer’s instruction. cDNA was obtained from total RNA using the TaqMan® MicroRNA Reverse Transcription kit and a mix of specific primers for miRNAs (Life Technologies), or the High-Capacity cDNA Reverse Transcription kit (Life Technologies) and random hexamers for all other genes. Gene expression analysis was performed by qPCR using TaqMan® hydrolysis probes (Life Technologies) specific for the *miR-204* (assay ID 000508), *miR-211* (assay ID 000514), *miR-9* (assay ID 000583), *miR-375* (assay ID 000564), insulin (*INS*) (assay ID Hs00356618_m1 or Hs02741908_m1), glucagon (*GCG*) (assay ID Hs01031536_m1), *PDX1* (assay ID Hs00426216_m1), *NKX6.1* (assay ID Hs00232355_m1)*, NKX2.2* (assay ID Hs00159616_m1)*, PAX4* (assay ID Hs00927346_g1)*, PAX6* (assay ID Hs00242217_m1)*, ONECUT1* (assay ID Hs00413554_m1)*, ISL1* (assay ID Hs00158126_m1)*, NGN3* (assay ID Hs00360700_g1)*, PTF1A* (assay ID Hs00603586_g1)*, NEUROD1* (assay ID Hs00139598_m1)*, TRPM3* (assay ID Hs00326297_m1), *TXNIP* (assay ID Hs01006900_g1), *MAFA* (assay ID Hs01651425_s1), and, as endogenous control the glyceraldehyde 3-phosphate dehydrogenase gene (*GAPDH*) (assay ID Hs99999905_m1). A commercial panel of total RNAs (FirstChoice, Life Technologies) was used for assessment of miRNA expression in other tissues. All qRT-PCR reactions were run for 40 cycles and samples that showed no amplification before the last cycle were defined as undetermined.

### Droplet digital PCR

*miR-204* and other genes’ transcript levels were investigated by droplet digital PCR (ddPCR). The ddPCR was performed on a QX100 ddPCR system (Bio-Rad, Hercules, CA, USA) using the previously described specific fluorescent hydrolysis probes and primers TaqMan assays. To avoid droplet saturation and achieve maximal RT efficiency the optimal amount of total RNA to be reverse transcribed and amplified by ddPCR was established for each transcript in preliminary experiments by testing sequential dilutions. The established optimal quantity for RT-ddPCR for each reaction corresponded to: 0.001 ng for *INS*, 5 ng for *MAFA* and *TXNIP*, 1 ng for *TRPM3*, 0.33 ng for *miR-204*, 0.25 ng for *GAPDH*. ddPCR reactions were emulsified in a QX100 droplet generator (Bio-Rad), transferred to 96 well plates and subjected to thermal cycling on a T100 instrument (Bio-Rad), according to the manufacturer’s instructions. After amplification, plates were read and individual sample droplets analyzed on a Bio-Rad QX100 droplet reader. The amount of target miRNA or gene mRNA copies per ng of analyzed total RNA was determined using the QuantaSoft v1.2.10 software, applying a correction based on the Poisson distribution to the counted number of droplets positive, and then taking into account the amount of reverse transcribed total RNA input in each reaction. An additional analysis of selected ddPCR results was also conducted by normalizing measured copies of target genes relative to those of the housekeeping gene GAPDH in the same samples.

### Assessment of c-peptide content in culture supernatant of EndoC-βH1 upon *miR-204* up- and down-regulation

Basal c-peptide levels in culture supernatants of EndoC-βH1 cells upon *miR-204* up- and down-regulation were measured using the Mercodia c-peptide ELISA kit (Mercodia AB, Uppsala, Sweden) following the manufacturer’s instructions. Differences in c-peptide levels across treatments were compared by calculating the percent change relative to the appropriate control reference.

### Assessment of transfected islet cell viability by flow cytometry

5 × 10^5^ islet cells from each transfection treatment were harvested after 72 hours and stained with the LIVE/DEAD™ Fixable Violet Dead Cell Stain Kit (Thermo Fisher Scientific). Intracellular staining required cell fixation (BD Cytofix/Cytoperm™, BD Biosciences, San Jose, CA, USA) and permeabilization with Perm Buffer III (BD Biosciences), following the manufacturer’s instructions. Cells were then stained with monoclonal Alexa Fluor® 647 Mouse Anti-Insulin antibody (Clone T56–706) (BD Biosciences) for 30 minutes at 4 °C. Analysis was carried out on a FACSCanto flow cytometer using FACS Diva software (BD Biosciences). Results were analyzed with the Cytobank platform (Cytobank, Inc., Santa Clara, CA, USA) and the percentage of live cells was calculated on the insulin positive cells.

### Bioinformatics analysis of *miR-204* target sequences in the *MAFA* 3’UTR

A bioinformatics analysis of potential mRNA targets of *miR-204* was performed with a panel of online tools based on alternative algorithms: miRanda (complementary, http://www.microrna.org), TargetScan (seed-complementary, http://www.targetscan.org), DIANAmicroT (thermodynamics, http://diana.imis.athena-innovation.gr/DianaTools/), RNAhybrid (Thermodynamics & Statistical model method, http://bibiserv.techfak.uni-bielefeld.de/rnahybrid), and MiRtaget2 (Support Vector Machine method, http://mirdb.org)^14–18^.

### Statistical analysis

Logistic regression was applied to the assessment of the association between miRNA levels and the endocrine phenotype. Differences in miRNAs levels between PET groups were assessed by the Mann-Whitney test. Correlation of *miR-204* and *miR-211* in PET samples was evaluated by linear regression. The statistical significance of changes in gene expression upon *miR-204* up-regulation was assessed using the Wilcoxon matched pair signed rank test. Changes in c-peptide levels across treatments were tested using one way anova and multiple comparisons. For all analyses, a 2-tailed *P* value of <0.05 was considered significant. Statistical analyses were performed using the Statistical Package for Social Science, Version 13.0 (SPSS) or Prism 6 (Graphpad software).

## Electronic supplementary material

Supplementary Information
