# Supplementary material for: miR-204 is associated with an endocrine phenotype in human pancreatic islets but does not regulate the insulin mRNA through MAFA
Source: Sci Rep. 2017 Oct 25;7:14051. doi: 10.1038/s41598-017-13622-7 (PMC5656581; doi:10.1038/s41598-017-13622-7)
Supplement: Supplementary file 1 — Supplementary Information [file 41598_2017_13622_MOESM1_ESM.pdf]

miR-204 is associated with an endocrine phenotype in human pancreatic islets but does not regulate the insulin mRNA through MAFA

Ilaria Marzinotto[1,2], Silvia Pellegrini[1], Cristina Brigatti[1], Rita Nano[1], Raffaella Melzi[1], Alessia Mercalli[1], Daniela Liberati[2], Valeria Sordi[1], Maurizio Ferrari[2,3], Massimo Falconi[3,4], Claudio Doglioni[3,5], Philippe Ravassard[6], Lorenzo Piemonti[1,3] *, Vito Lampasona[2]*

(1) Diabetes Research Institute, Division of Immunology, Transplantation and Infectious Disease, IRCCS San Raffaele Scientific Institute, Milan, Italy

(2) Human Pathologies Genomic Diagnostics unit, Division of Genetics and Cell Biology, San Raffaele Scientific Institute, Milan, Italy

(3) Vita-Salute San Raffaele University, Milan, Italy

(4) Department of Surgery, Division of Pancreatic Surgery, San Raffaele Scientific Institute, Milan, Italy

(5) Unit of Pathology, San Raffaele Scientific Institute

(6) Institut du Cerveau et de la Moelle épinière (ICM), Biotechnology & Biotherapy Team, Université Pierre et Marie Curie, Paris, France

Supplementary Table 1 Description of pancreatic endocrine tumors

Classification[a]	WHO classification[b]	Patient Sex/Age	Size (cm)	Pancreas site	Pr.Index[c]	Functional	Insulin[d]	Glucagon[d]	Somatostatin[d]	Pancreatic Polipeptide[d]
Ins-F-PET	WDET (uncertain behavior)	Male/31	2.3	Body/tail	1.0%	Yes	100%	Neg	Neg	Neg
Ins-F-PET	WDET (uncertain behavior)	Male/63	2.8	Head	2.0%	Yes	90%	Neg	Neg	Neg
Ins-F-PET	WDET (benign)	Female/68	1.4	Body/tail	1.0%	Yes	95%	Neg	Neg	Neg
Ins-F-PET	WDEC	Male/65	3.5	Body/tail	7.5%	Yes	5%	5%	Neg	Neg
Ins-F-PET	MEN1	Female/32	2.5	Body/tail	1.0%	Yes	20%	60%	Neg	Neg
Ins-F-PET	WDET (uncertain behavior)	Female/47	1.6	Body/tail	1.0%	Yes	90%	Neg	Neg	Neg
Ins-F-PET	MEN1	Male/44	1.0	Body/tail	2.0%	Yes	90%	Neg	Neg	Neg
Gluc/Som-F-PET	WDET (benign)	Male/55	1.8	Body/tail	1.0%	Yes	Neg	95%	80%	10%
Gluc/Som-F-PET	WDET (uncertain behavior)	Female/46	2.3	Body/tail	1.0%	Yes	Neg	10%	Neg	Neg
Gluc/Som-F-PET	WDET (uncertain behavior)	Female/76	3.5	Head	4.0%	Yes	Neg	Neg	5%	Neg
Gluc/Som-F-PET	WDET (uncertain behavior)	Male /82	4.0	Body/tail	4.0%	Yes	Neg	30%	Neg	Neg
NF-PET	WDET (uncertain behavior)	Female/50	1.7	Body/tail	1.0%	No	Neg	Neg	Neg	Neg
NF-PET	WDET (uncertain behavior)	Female/19	4.7	Body/tail	4.0%	No	Neg	Neg	Neg	Neg
NF-PET	WDEC	Female/58	10.0	Body/tail	4.0%	No	Neg	Neg	Neg	Neg
NF-PET	WDEC	Male/62	2.0	Body/tail	5.0%	No	Neg	Neg	Neg	Neg
NF-PET	WDET (benign)	Male/36	1.0	Body/tail	0.5%	No	Neg	Neg	Neg	Neg
NF-PET	WDET (benign)	Female/68	1.0	Body/tail	1.0%	No	Neg	Neg	Neg	Neg
NF-PET	WDEC	Male/41	2.3	Head	3.0%	No	Neg	Neg	Neg	Neg

[a]Ins-F-PET: Insulinomas; Gluc/Som-F-PET: Glucagonomas or Somatostatinomas; NF-PET: Non Functional Pancreatic Endocrine Tumors

[b]WDET: well-differentiated endocrine neoplasms; WDEC: well-differentiated endocrine carcinomas; MEN1: Multiple endocrine neoplasia type 1

[c]Pr. Index = proliferative index based on evaluation of Ki67

[d]percentage of cells positive for the protein in immunohystochemistry

Supplementary Table 2: Linear regression analysis of selected miRNAs and pancreatic transcription factors gene expression and its respective statistical significance in PETs

	miR-204		*miR-211*		*miR-375*		*miR-9*	
	R^2	P	R^2	P	R^2	P	R^2	P
PDX1	0.0001	0.97	0.0043	0.84	0.0005	0.95	0.0005	0.94
NKX6.1	0.0027	0.87	0.0121	0.73	0.0013	0.91	0.0004	0.95
NKX2.2	0.2321	0.19	0.1141	0.37	0.0086	0.83	0.0277	0.67
PAX4	0.3336	0.17	0.4032	0.18	0.0324	0.70	0.2962	0.21
PAX6	0.0001	0.98	0.0052	0.82	0.3196	0.06	0.1164	0.25
ONECUT1	0.0040	0.87	0.0001	0.98	0.0680	0.53	0.0213	0.71
ISL1	0.0219	0.63	0.0954	0.33	0.0075	0.79	0.1245	0.24
NGN3	0.0418	0.63	0.0103	0.83	0.0000	0.99	0.0223	0.72
PTF1A	0.0421	0.66	0.0358	0.72	0.5013	0.12	0.1635	0.37
NEUROD1	0.0263	0.65	0.0000	1.00	0.0055	0.85	0.0009	0.93

Supplementary Figure 1

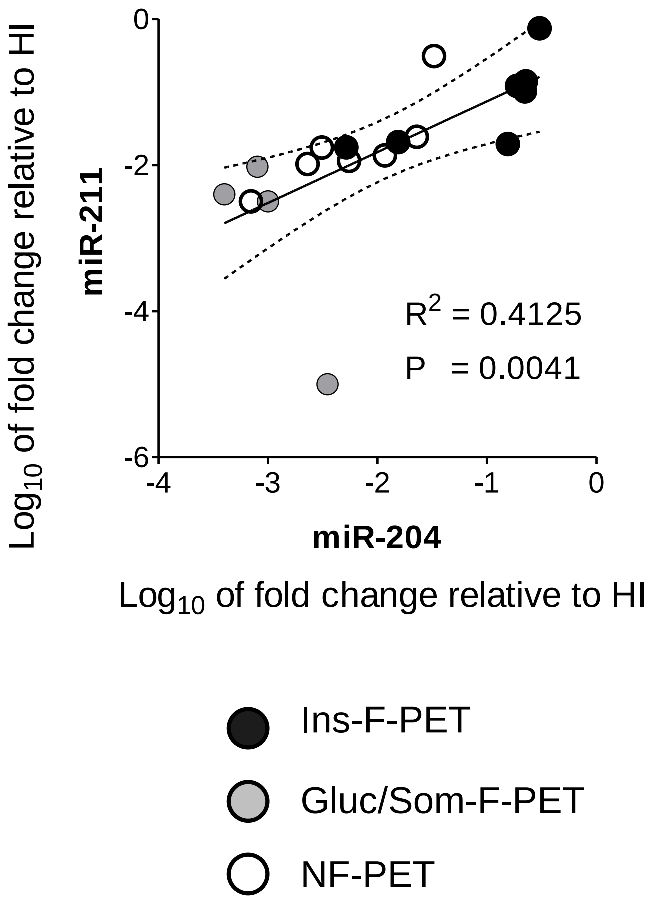

Correlation of *miR-204* and *miR-211* expression in NET. XY plot of *miR-204* and *miR-211* levels in Ins-F-PET (black circles), Gluc/Som-F-PET (grey circles), and NF-PET (clear circles) evaluated by qRT-PCR and expressed as fold change relative to median levels in human islets (HI); correlation was evaluated by linear regression, dotted lines represent the 95% confidence interval.

Supplementary Figure 2

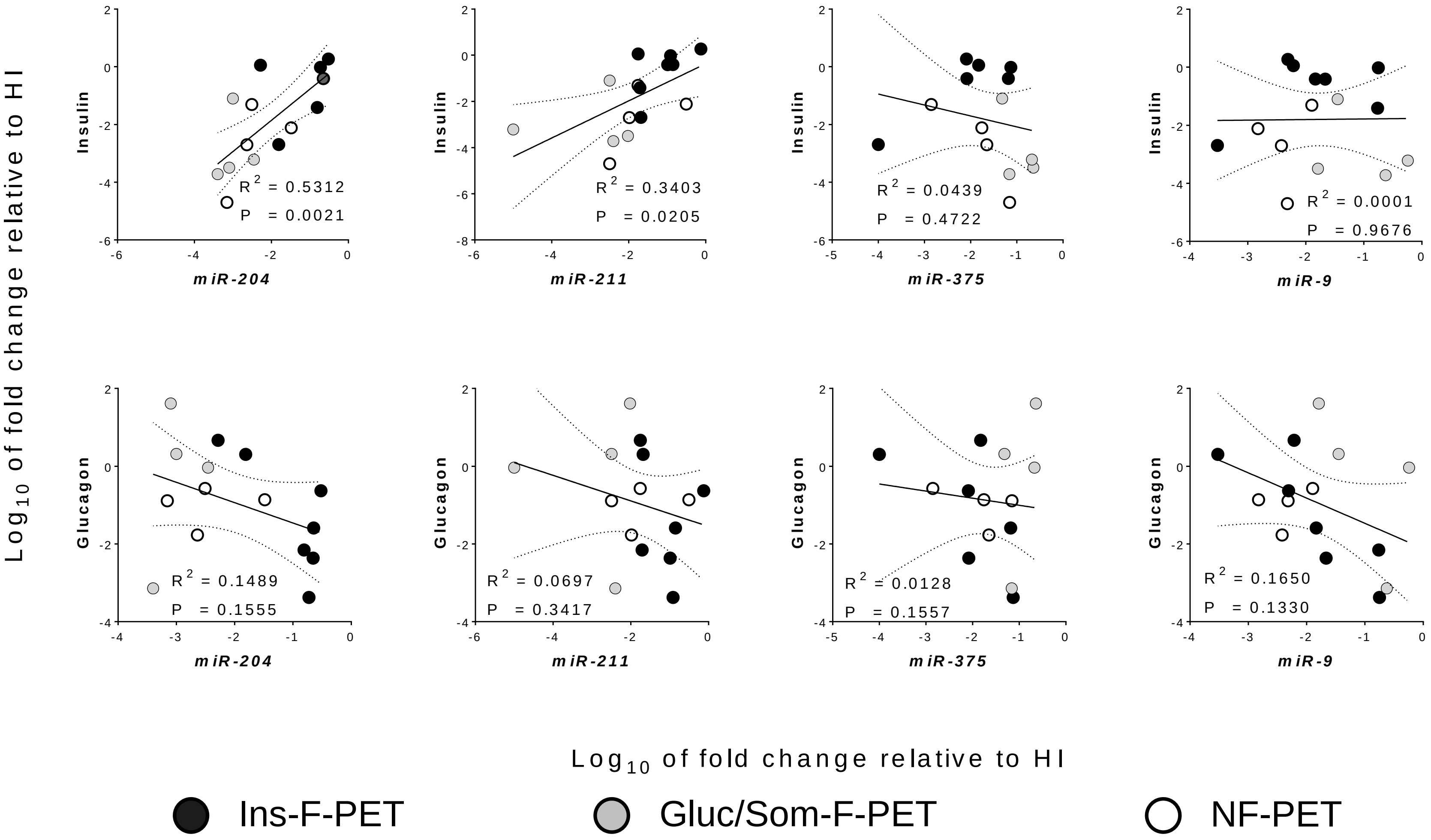

Correlation of selected miRNA expression with either insulin or glucagon mRNA in PET. XY plot of *miR-204*, *miR-211*, *miR-375*, and *miR-9* vs insulin (top panels) or glucagon (lower panels) mRNAs evaluated by qRT-PCR and expressed as fold change relative to median levels in human islets (HI). Samples include: Ins-F-PET (black circles), Gluc/Som-F-PET (grey circles), and NF-PET (clear circles). Correlation was tested by linear regression, dotted lines represent the 95% confidence interval.

Supplementary Figure 3

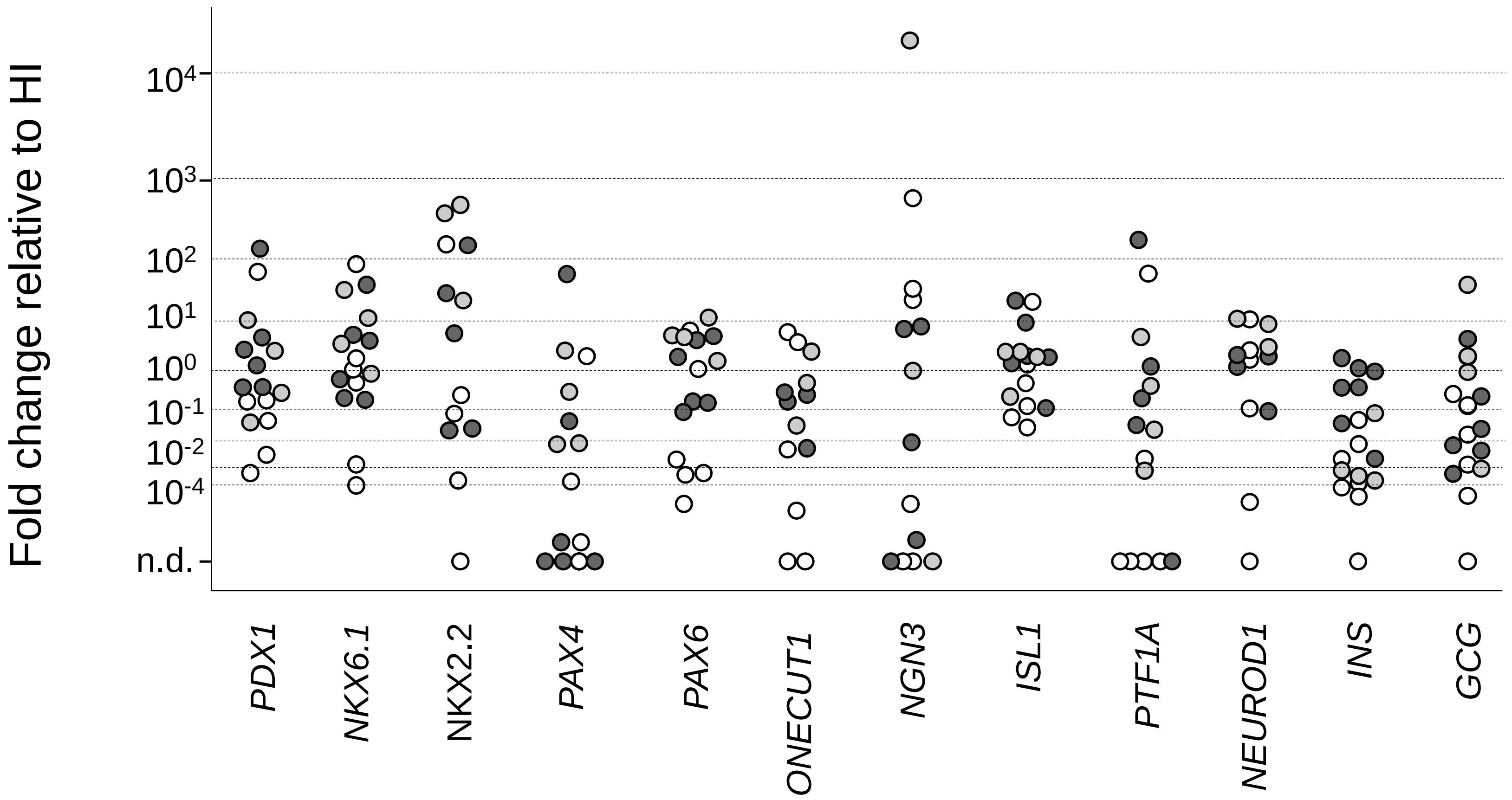

mRNA expression in PETs of transcription factors involved in pancreatic development. Levels of *PDX1*, *NKX6.1*, *NKX2.2*, *PAX4*, *PAX6*, *ONECUT1*, *NGN3*, *ISL1*, *PTF1A*, *NEUROD1*, as well as those of insulin (INS) and glucagon (GCG), as β or α cells markers, were determined using qRT-PCR. Values are reported as fold change relative to median expression in human islets (HI). Ins-F-PET, dark grey circles; Gluc/Som-F-PET, light grey circles; NF-PET, clear circles; n.d. = not detected.

Supplementary Figure 4

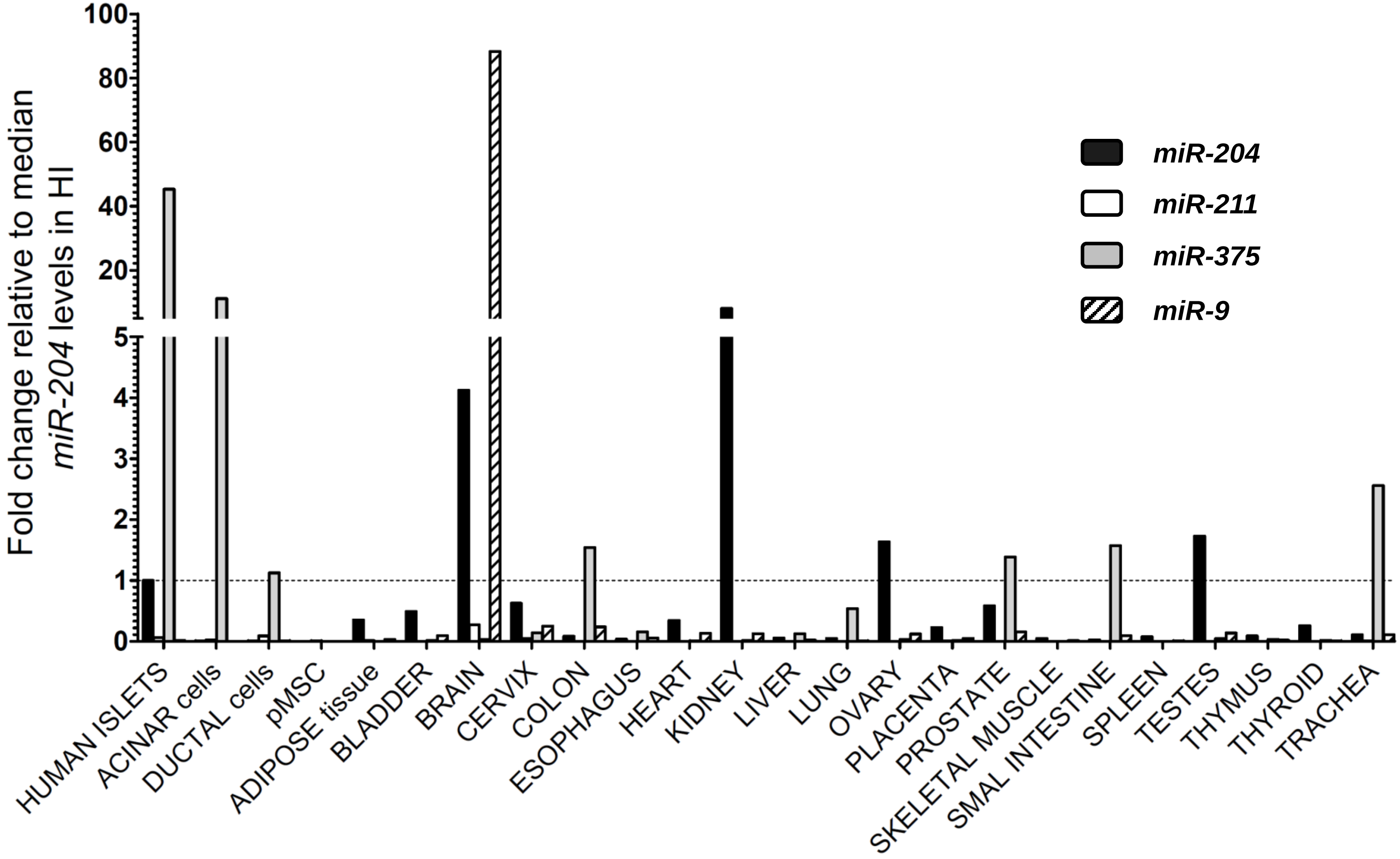

Selected pancreatic miRNA expression in a panel of human tissues. Bar plot of *miR-204* (black bars), *miR-211* (clear bars), *miR-375* (grey bars), and *miR-9* (striped bars) levels in human islets (HI), acinar, ductal, pMSC cells and in a panel of human tissues. Expression was measured by qRT-PCR and is reported as fold change relative to median miR-204 levels in HI (dotted line).

Supplementary Figure 5

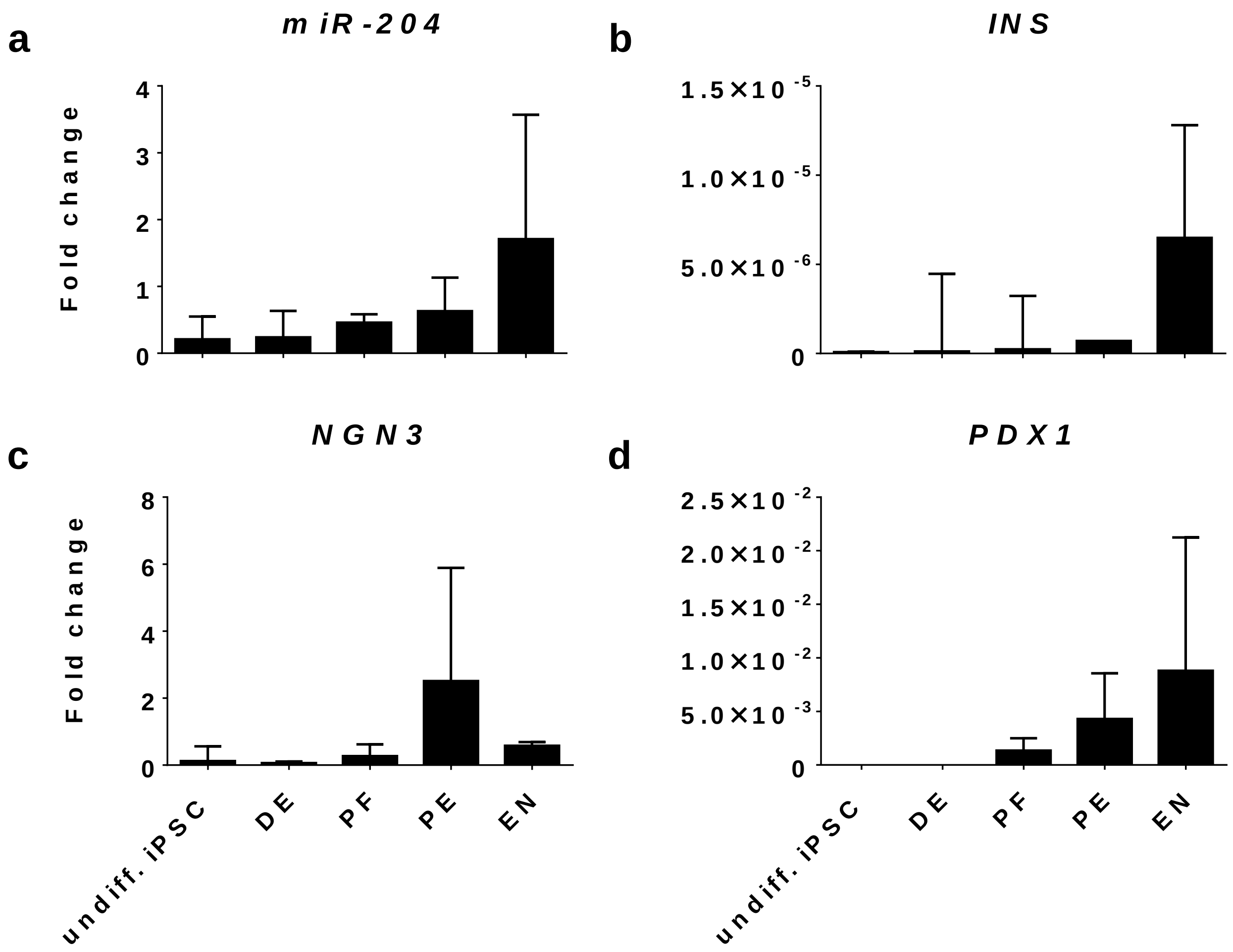

Expression of *miR-204*, *INS*, *NGN3*, and *PDX1* mRNAs in the course of differentiation of human fetal fibroblasts derived iPSCs towards a β cell-like phenotype.

Bar plot of mRNA fold change (mean+SD) relative to median expression in human islets (HI) as measured by qRT-PCR at five sequential differentiation stages: undifferentiated iPSCs (undiff. iPSC); definitive endoderm (DE); posterior foregut (PF); pancreatic endoderm (PE); hormone expressing endocrine cells (EN).

Supplementary Figure 6

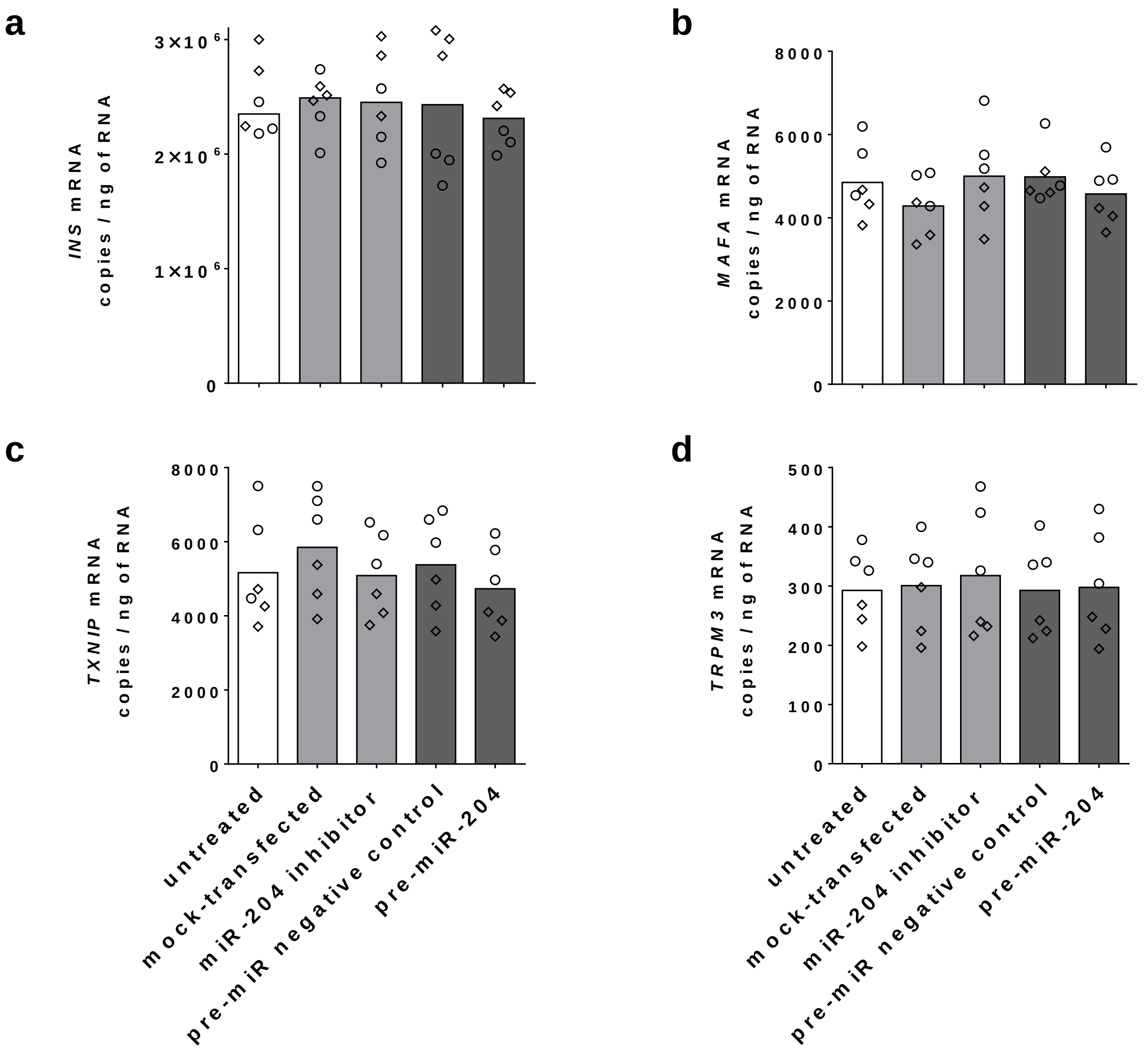

Effect of *miR-204* up- or down-regulation on *INS*, *MAFA*, *TXNIP*, and *TRPM3* transcripts in preparations of isolated human islets 72 hours post transfection.

Bar plots of average copies per ng of total RNA measured by ddPCR for the *INS*, *MAFA*, *TXNIP*, and *TRPM3* (panel a, b, c, d, respectively) in *miR-204* up- and down-regulation experiments. Overlaid are the individual results of 3 transfection performed in parallel on two different batches of purified human islets (Circles=HP1266; diamonds=HP1269). Treatment groups are identified by bar color: untreated cells white bars, *miR-204* down-regulation light grey bars, *miR-204* up-regulation dark grey bars.

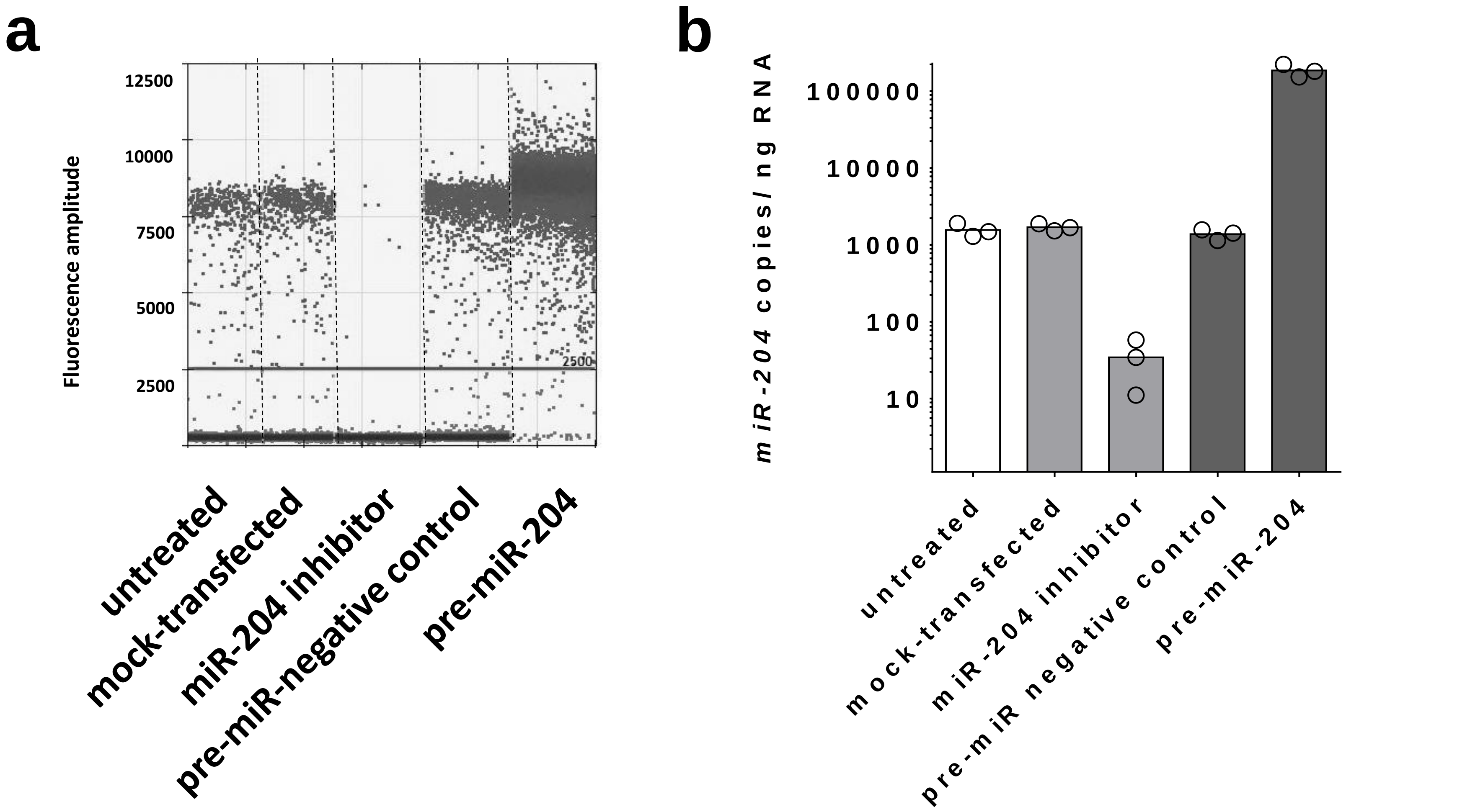

***miR-204* up- and down-regulation in EndoC-βH1 cells 72 hours post transfection.** Scatter plot of ddPCR droplets' fluorescence results from a representative experiment of miR-204 up- or down-regulation in EndoC-βH1 cells. Vertical dotted lines separate treatment groups (panel a). Bar plot of mean *miR-204* copies per ng of total RNA measured by ddPCR, according to treatment group. Individual results of each transfection replicate (n=3) are shown as circles. Treatment groups are identified by bar color: untreated cells white bars, *miR-204* down-regulation light grey bars, *miR-204* up-regulation dark grey bars (panel b).

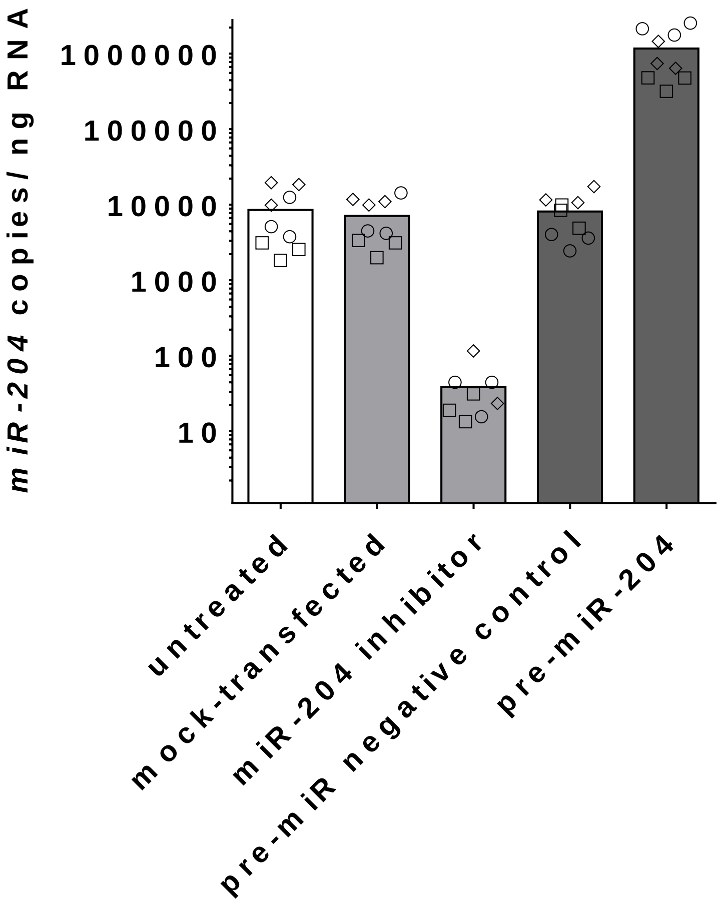

miR-204 up- and down-regulation in EndoC-βH1 cells 48 hours post transfection.

Bar plot of mean *miR-204* copies per ng of total RNA measured by ddPCR in all experiments, according to treatment group. Overlaid are the individual results of each transfection (n=3) performed in parallel in each independent experiment (squares=experiment 1; circles= experiment 2; diamonds=experiment 3). Treatment groups are identified by bar color: untreated cells white bars, *miR-204* down-regulation light grey bars, *miR-204* up-regulation dark grey bars.

Supplementary Figure 9

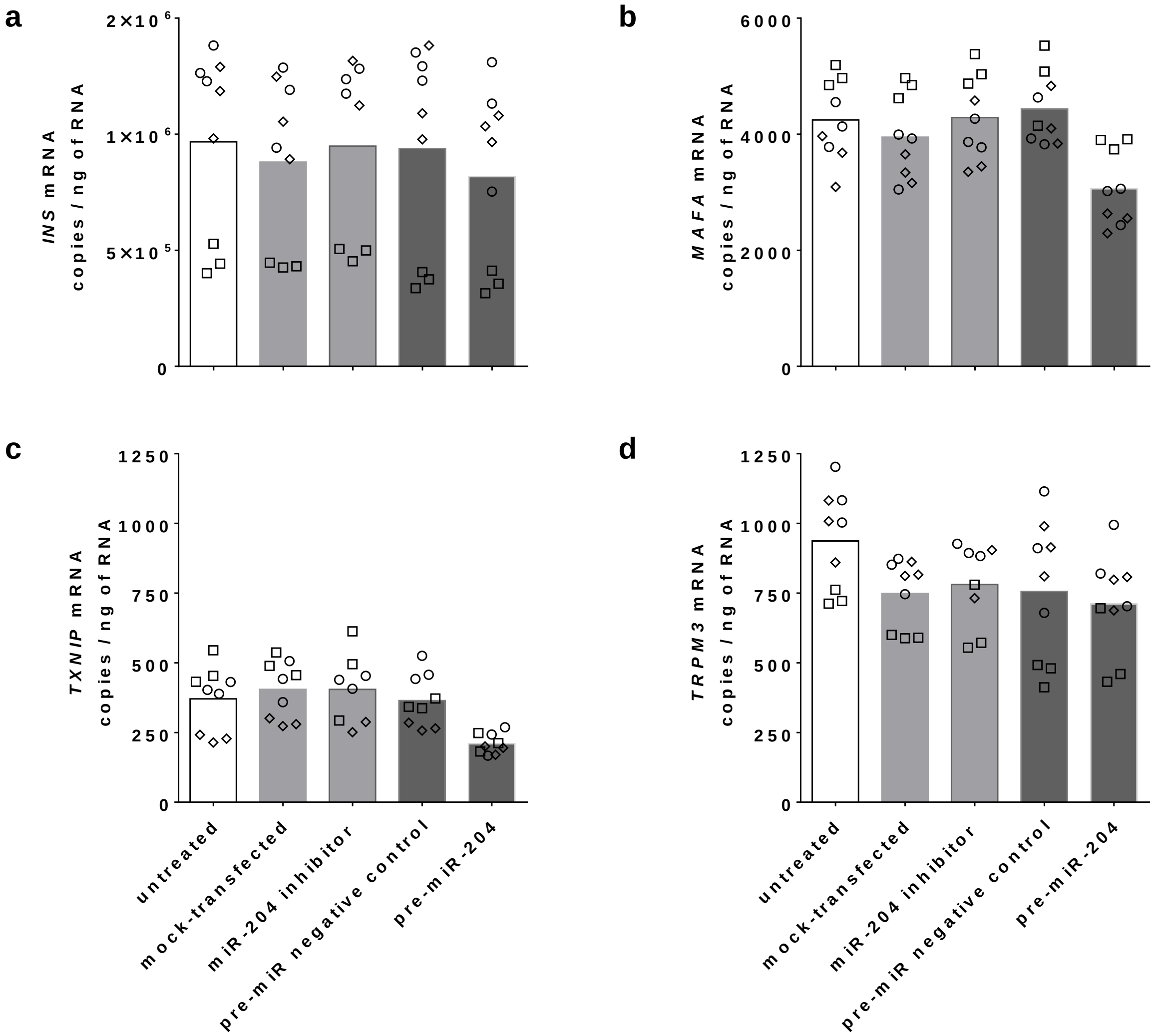

Effect of *miR-204* up- or down-regulation on *INS*, *MAFA*, *TXNIP*, and *TRPM3* transcripts in EndoC-βH1 48 hours post transfection.

Bar plots of average copies per ng of total RNA measured by ddPCR for the *INS*, *MAFA*, *TXNIP*, and *TRPM3* (panel a, b, c, d, respectively) in *miR-204* up- and down-regulation experiments. Overlaid are the individual results of each transfection (n=3) performed in parallel in each independent experiment (squares=experiment 1; circles= experiment 2; diamonds=experiment 3). Treatment groups are identified by bar color: untreated cells white bars, *miR-204* down-regulation light grey bars, *miR-204* up-regulation dark grey bars.

Supplementary Figure 10

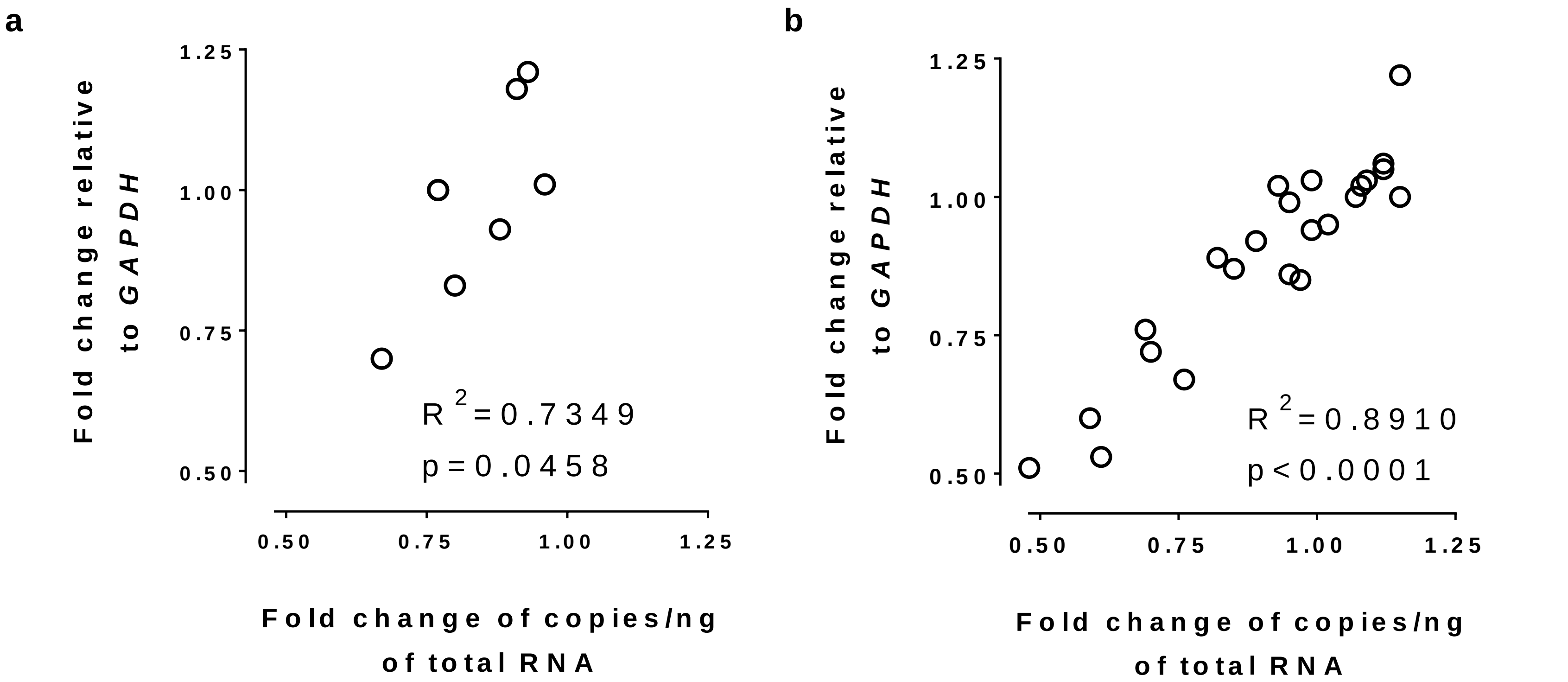

Correlation of mRNAs fold changes with or without normalization of copy number relative to the levels of the *GAPDH* transcript. XY plot of the fold change of *INS*, *MAFA*, *TXNIP*, and *TRPM3* transcripts upon *miR-204* up- and down-regulation are shown for the 72 hours (panel a) and 48 hours (panel b) transfections. On the x axis is plotted the fold change of the measured copies per ng of total RNA in treated samples relative to the appropriate control samples. On the y axis is plotted the fold change of measured copies after normalization relative to the *GAPDH* transcript level also measured by ddPCR in the same samples. Shown are the corresponding Spearman R^2 and p values.

Supplementary Figure 11

Rat MAFA 3’UTR	CCCGAGAA**C**GGU**G**A**AU**ACUU**AAGGAA**GCC
rno-miR-204	UCC**G**UAU**C**C**UA**CUGU**UUCCCUU**
Human MAFA 3’UTR	AGCGGA**G**AACGGU**GAU**UU**C**T**AAGG**A**AA**CTT
hsa-miR-204	U**C**CGUAUC**CUA**CU**G**U**UUCC**C**UU**

Alignment of human and mouse *miR-204* and its putative 3’ UTR of the *MAFA* target sequence. Mature *miR-204* sequences from Rat (NCBI Refseq NR_031919.1) and human (NCBI Refseq NR_029621.1) were aligned against their candidate target sequence within the 3’ UTR of the MAFA transcript of their respective species (NCBI Refseq XM_006241903.2 and NM_201589.3). Nucleotides matching between *miR-204* and MAFA 3’ UTR are highlighted in bold and underlined, the putative seed sequence is shown in red.
